# Genomics of sablefish (*Anoplopoma fimbria*): expressed genes, mitochondrial phylogeny, linkage map and identification of a putative sex gene

**DOI:** 10.1186/1471-2164-14-452

**Published:** 2013-07-06

**Authors:** Eric B Rondeau, Amber M Messmer, Dan S Sanderson, Stuart G Jantzen, Kristian R von Schalburg, David R Minkley, Jong S Leong, Graham M Macdonald, Amanda E Davidsen, William A Parker, Rosetta SA Mazzola, Briony Campbell, Ben F Koop

**Affiliations:** 1 Department of Biology, Centre for Biomedical Research, University of Victoria, Victoria, British Columbia V8W 3N5, Canada; 2 Sablefish Canada Ltd, 335 Walkers Hook Rd., Salt Spring Island, British Columbia V8K 1N7, Canada

**Keywords:** Sablefish, Black cod, Microsatellite, SNP, Linkage map, Conserved synteny, Threespine stickleback, Sex-specific sequences, Gonadal soma-derived factor

## Abstract

**Background:**

The sablefish (order: Scorpaeniformes) is an economically important species in commercial fisheries of the North Pacific and an emerging species in aquaculture. Aside from a handful of sequences in NCBI and a few published microsatellite markers, little is known about the genetics of this species. The development of genetic tools, including polymorphic markers and a linkage map will allow for the successful development of future broodstock and mapping of phenotypes of interest. The significant sexual dimorphism between females and males makes a genetic test for early identification of sex desirable.

**Results:**

A full mitochondrial genome is presented and the resulting phylogenetic analysis verifies the placement of the sablefish within the Scorpaeniformes. Nearly 35,000 assembled transcript sequences are used to identify genes and obtain polymorphic SNP and microsatellite markers. 360 transcribed polymorphic loci from two sablefish families produce a map of 24 linkage groups. The sex phenotype maps to sablefish LG14 of the male map. We show significant conserved synteny and conservation of gene-order between the threespine stickleback *Gasterosteus aculeatus* and sablefish. An additional 1843 polymorphic SNP markers are identified through next-generation sequencing techniques. Sex-specific markers and sequence insertions are identified immediately upstream of the gene gonadal-soma derived factor (*gsdf*), the master sex determinant locus in the medaka species *Oryzias luzonensis*.

**Conclusions:**

The first genomic resources for sablefish provide a foundation for further studies. Over 35,000 transcripts are presented, and the genetic map represents, as far as we can determine, the first linkage map for a member of the Scorpaeniformes. The observed level of conserved synteny and comparative mapping will allow the use of the stickleback genome in future genetic studies on sablefish and other related fish, particularly as a guide to whole-genome assembly. The identification of sex-specific insertions immediately upstream of a known master sex determinant implicates *gsdf* as an excellent candidate for the master sex determinant for sablefish.

## Background

The order Scorpaeniformes is a diverse group of species that include the rockfish, greenling, sculpins and sablefish among others. Grouped originally by the presence of the suborbital stay, a posterior extension of the third circumorbital bone [1], the order is now considered to be paraphyletic with members of the orders Perciformes and Gasterosteiformes [2]. While a number of species among the currently defined order are considered commercially important, the most economically valuable in North America is the sablefish, *Anoplopoma fimbria*.

The sablefish, also known as Alaskan black cod, is a long-lived demersal species located mainly between 200-1500 m along the continental shelf of the North Pacific Ocean [3]. While found from eastern Japan through Alaskan waters and down to Baja California in Mexico [4], the sablefish is most important to American and Canadian commercial fisheries, with sablefish landings exceeding $200 million value on a combined harvest of 22,000 metric tonnes in 2011 [5,6]. Sablefish is also in the preliminary stages of commercial aquaculture, with farms in British Columbia already producing 500 tonnes in 2009 [7]. While commercially important, little genetic information is available, with genetic resources limited to a few sequences and 29 microsatellite primer pairs [8-10]. Indeed little is known about the 1,477 species [11] within the economically important order Scorpaeniformes.

For the successful development of sablefish as a sustainable species in aquaculture and the protection of wild fisheries stocks, modern molecular tools could be of great value. The identification and exploitation of genetic markers can be used in the characterization and identification of strains, parental identification and analysis of diversity in the broodstock or in the construction of a linkage map [12]. Linkage maps have been developed for numerous fish species including Atlantic salmon [13,14], channel catfish [15], common carp [16], grass carp [17], Atlantic halibut [18] and gilthead sea bream [19]. While traditionally maps were developed with markers such as allozymes, AFLP and RAPD markers, microsatellites and SNPs are the current markers of choice. Microsatellites are relatively abundant, highly polymorphic and easy to genotype, and SNPs, while less informative due to a limit of two (or very occasionally three) alleles per locus, are easily identifiable and are the marker of greatest abundance in the genome. With advances in next-generation sequencing (NGS) protocols such as RAD mapping [20] and Genotyping-by-Sequencing [21] it is possible to generate a dense SNP map of primarily anonymous markers (Type II markers) with relatively little prior DNA sequence information. Having markers linked to genes (Type I markers) rather than anonymous sequences, however, allows for the putative placement of genes on a linkage map, which can make them of greater use than type II markers in linkage mapping for aquaculture species [12]. Type I markers, both microsatellites and SNPs, can easily be identified in libraries of expressed sequences, either through traditional EST library sequencing or through NGS transcript sequencing methods such as RNAseq. While linkage maps can have many uses, one of the most useful is in mapping phenotypes to a map in an effort to identify the genes controlling phenotype and look for associations or linkage between traits. For single locus phenotypes, the phenotype can be scored and analyzed in the way a genetic marker would; multi-locus phenotypes require more powerful quantitative trait loci (QTL) analyses to statistically predict regions of interest.

Of the phenotypes often identified through mapping, sex is one of the most important and most common for many species. Sexual dimorphism is of particular significance as one sex can often grow larger or faster, both important traits to understand in fisheries and aquaculture. While mapping of the sex phenotype to a chromosome is relatively easy given a single-locus sex determination system, mapping the sex phenotype to a gene has been more difficult, and the master sex determinant has only been identified in a few fish species. *Dmy* determines sex in medakas *Oryzias latipes*[22] and *O. curvinotus*[23], *amhy* in the Patagonian pejerrey *Odontesthes hatcheri*[24], and *sdY* in most salmonids [25,26]. These master sex determining genes (MSD) are the result of divergent duplicated copies of autosomal genes, present only on the Y-chromosome. *Gsdf*^Y^ in the medaka *Oryzias luzonensis,* on the other hand, is up-regulated in males during sexual differentiation, due to changes in the upstream promoter region [27], while the male-specific *Amhr2*^*Y*^ in 3 species of *Takifugu* appears to be the result of a single coding change [28]. While all these genes have been previously described as playing a role in sexual determination (aside from *sdY*), none so far have been described as the master sex determinants in distantly related groups of fish.

Comparative mapping [29] can be used to facilitate comparison of newly developed maps to pre-existing genomic resources to augment available genetic information. Comparison between non-model fish species and those with a fully-sequenced reference genome has identified significant conserved synteny in numerous species [15,17,30-32]. With preliminary or complete whole-genome assemblies available for ten bony fish species (October 28, 2012) [33], and the increasing ease with which genetic information and complete genomic sequences can be obtained, comparative mapping will only become easier. The degree to which the identification of conserved synteny will be useful depends on how closely related the two species are; thus, for species of relatively close ancestry and significant macro-synteny, comparative mapping can be used to predict gene locations and order, and provide a list of potential candidates responsible for a particular phenotype or underlying a QTL.

In this work, we describe the sequencing of the mitochondrial genome and the resultant verification of the phylogenetic placement of the sablefish within the Scorpaeniformes. We present a library of assembled transcript sequences to identify genes, and use them to develop type I polymorphic SNP and microsatellite markers. The markers were scored across two families and used to produce the first-generation sablefish linkage map and locate the sex phenotype onto the male map. We show significant conserved synteny between the threespine stickleback *Gasterosteus aculeatus* and sablefish, and use a comparative mapping approach to predict gene locations in the sablefish. An additional collection of markers, mainly type II SNPs are identified through genotyping-by-sequencing and used to identify sex-specific markers and sequence insertions immediately upstream of a known master sex determinant.

## Results and discussion

### Gene identification

In order to obtain a comprehensive picture of the gene sequences of sablefish, we examined transcriptomes by EST sequencing and RNAseq. In total, 19,968 cDNA clones were sequenced in both forward and reverse directions. Following trimming and removal of any contaminants, 34,080 EST sequences were obtained, and deposited in the GenBank EST database under accessions GenBank:GO615858-GO649937. EST sequences were assembled into 12,060 unique contigs using PHRAP assembly. 1,249 full length gene sequences were submitted as GenBank:ACQ57837–ACQ59081, GenBank:C3KHG1, GenBank:C3KJF2, GenBank:C3KHF2 and GenBank:C3KJE6. A 3’ UTR analysis of all contigs was used for primer design in the search for polymorphic markers. RNAseq yielded 96,733,584 reads, which were assembled into 92,888 Unigene sequences. Combined with the EST sequencing data into NCBI TSA BioProject 71237, the assembled data produced 34,728 contigs >400bp; these can be found in accession numbers GenBank: JO657891-JO692618.

The uploaded EST and TSA datasets represent all but 84 of the 70,138 sequences for sablefish in the NCBI nucleotide and EST databases (retrieved October 7, 2012). In addition, sablefish is currently the Scorpaeniformes species with the largest percentage of sequences in both the EST (46.9% of total) and nucleotide databases (72.7%). This work represents a very significant increase in the available expressed sequence data for the sablefish, as well as for the order Scorpaeniformes in general.

### The mitochondrial genome and phylogenetic placement of sablefish

In order to help resolve the general phylogenetic placement of sablefish we sequenced its mitochondrial genome and used a phylogenetic tree analysis to compare it to other Percomorpha and Scorpeaniformes. The sablefish mitochondrial genome, seen in Additional file 1: Figure S1, consists of 16,507 base pairs, encompassing 13 genes, 2 ribosomal RNAs and 22 tRNAs, with all features following the order of the typical vertebrate mitochondrial genome (reviewed in [34]). The sequence was submitted to NCBI as GenBank: JX070112.

**Figure 1:**
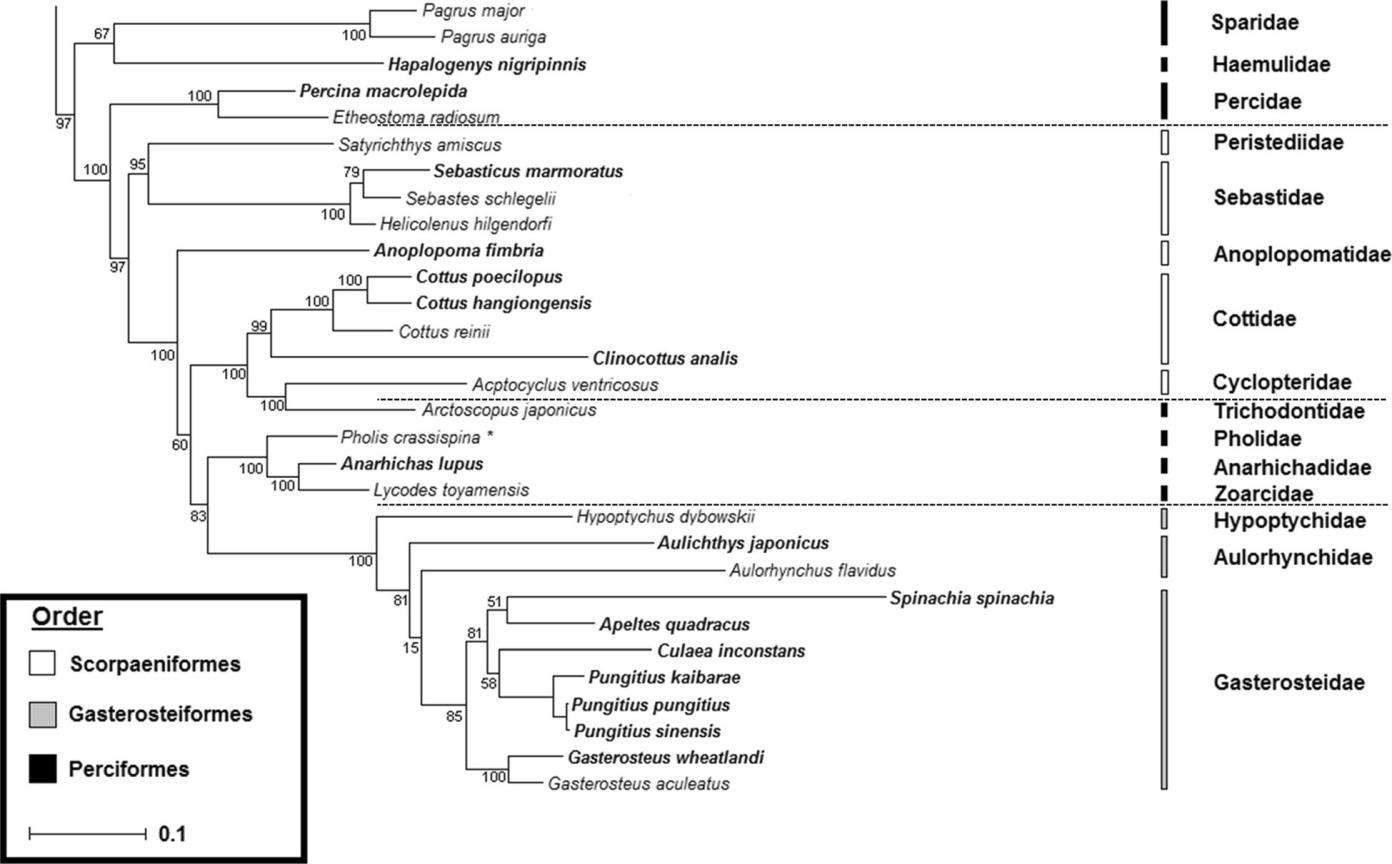
**Mitogenome phylogeny of orders: Scorpaeniformes, Gasterosteiformes, select Perciformes.** Species represented in bold text are not part of the previous analysis by Kawahara et al. [35]. Families currently assigned to the order Scorpaeniformes are represented by white bars down the right hand side, while Perciformes and Gasterosteiformes are in black and grey respectively. Boot-strap values are indicated for all branch points. The asterix (*) indicates a change of genus name from previous analyses.

The phylogenetic analysis of the mitogenome sequences produced the same major phylogenetic groupings as in Kawahara et al. [35], so we focused on the grouping of Scorpaeniformes, Zoarcoidei, and Gasteroidei. As seen in Figure 1, we recovered a very similar set of phylogenetic placements for the species previously analyzed (Subgroup G in [35]). Additionally, we recovered a similar phylogenetic ordering of the Gasterosteiformes mitogenomes [36]. The sablefish mitogenome was placed in an ancestral branch prior to the split of the Scorpaeniformes family Cottidae from the most-closely related Gasterosteiformes and Perciformes families, although the bootstrap value of this placement is not strong.

The Anoplopomatidae is a family comprising two known species, the sablefish and the skilfish (*Erilepis zonifer*) placed in the order Scorpaeniformes. Molecular ([1]; Figure 1) and morphological [37] analyses both suggest a closer relationship between sablefish and the cottoids than to the scorpanoids, but the exact placement remains unclear. While our analysis agrees with that of Smith and Wheeler [1] in suggesting a relatively ancestral branch for the divergence of the sablefishes, our results disagree on whether divergence occurred before or after (respectively) the divergence of the Cottoids from the Gasterosteodei and the Zoarcoidei; both analyses produce weak branch support in favour of the respective positions. Given the difficulty in accurately placing deep phylogenetic branches, a definitive molecular placement of the Anoplopomatidae will await further data.

While the exact placement of the Anoplopomatidae remains to be determined, sablefish represent an older unique branch that split at or around the split of Cottoidei and Gasterosteodei. This is of particular interest as one member of the Gasterosteodei, the threespine stickleback *Gasterosteus aculeatus,* has a fully sequenced draft genome available for comparison. The stickleback is without question the most closely related species for which a well annotated whole genome is available. Previous estimates based on mitochondrial genomes place the time of divergence of the more ancestral suborder Scorpaenoidei from the Cottoidei and Gasterosteodei at approximately 150 mya [38], meaning the divergence of sablefish from stickleback lineages could be less than 150 mya. More recent estimates using nuclear data suggest a much more recent time of divergence in the range of 50–70 mya [39]. The next most closely related species with fully sequenced genomes, those of the pufferfishes *Takifugu rubripes* and *Tetraodon nigroviridis* are separated from sablefish by 80–180 mya [38,39]. As all the phylogenetic data points to the stickleback as the closest fully-sequenced genome, *G. aculeatus* was used in our analysis of conserved synteny.

### Identification of genetic markers

A set of polymorphic microsatellite and SNP DNA markers from our EST assemblies was identified for use in genetic mapping and population studies. As the EST library construction and subsequent primer design was performed prior to obtaining sablefish family tissues, the sequences do not represent either of the two families used in linkage map construction. This did not affect the use of microsatellites as the identification and amplification of polymorphic repeats proved relatively easy, with over half the 460 primer pairs designed successfully amplifying the desired product and yielding polymorphic repeats in one or both sablefish families. In contrast, the identification of SNPs from the assembled EST dataset proved much less useful when targeting expected single base polymorphisms in our mapping families. However, in analyzing the adjacent sequence, mainly the 3’ UTR, it was found that sablefish were indeed highly polymorphic. This led to the strategy of identifying SNPs through the direct sequencing of 250 unrelated 3’ UTR gene regions of the sablefish family parents. Additional primers were designed to genes of interest based on conserved sequence identified in related species. Primer pair sequences, repeat motifs and other relevant information for the 233 newly identified microsatellites as well as the 13 previously published [8] can be found in Additional file 2: Table S1. For the SNPs, primer and annotation information can be found in Additional file 3: Table S2, with individual SNPs identified in Additional file 4: Table S3.

A preliminary set of markers was used to analyze a second, half-sibling family to determine the number of progeny from each of the three fathers. The majority of the progeny were descended from a single father (96 offspring) with only minor contributions from the other two fathers (13 and 6). It was therefore decided to analyze family 2 as a single full-sib family, excluding the handful of individuals descended from the other two fathers. Following genetic marker identification, we began locating these markers in two sablefish families.

### Production of first-generation linkage map

Equipped with our gene-associated markers and two sablefish families, we examined all of the markers in all the individuals from the two families to create a first-generation linkage map. In all, 246 microsatellites and 509 SNPs were scored for all of the individuals from the two families. When it came to assembling the linkage map, the strategy of identifying multiple SNPs in a single sequence proved very useful. While most of the microsatellite markers were highly polymorphic and useful for mapping in both families, it was very rare that individual SNPs were shared between families. It was common, however, to have two or more markers from a single EST assembly-derived sequence polymorphic in separate parents and families. As these markers are physically linked, separated by 600bp or less in a single contiguous sequence, we analyzed the SNPs as representing the locus rather than a particular marker and placed the locus on the linkage map rather than the individual nucleotide marker. This strategy was also used when multiple repeats designed from the same contig were analyzed. This allowed merging of the individual parental maps and sex-specific maps at many more sites than with the microsatellites alone, leading to a more accurate representation of marker order and distances. The 509 scored SNPs, therefore, were mapped as 133 SNP loci; between 1 and 13 SNPs contributed to mapping of an individual locus.

We produced maps for each of the mapping parents, with subsequent integration into sex-specific maps and finally a merged map. The final merged map, seen in Additional file 5: Figure S2, consists of all 133 SNP loci and 227 microsatellite loci (234 individual microsatellites) mapped across 24 linkage groups. Two additional microsatellite markers, AfiMI0079UVic and AfiMI0131UVic, remain unlinked and 10 primer pairs, while polymorphic, were uninformative in both mapping families (AfiMI0005UVic, AfiMI0015UVic, AfiMI0104UVic, AfiMI0153UVic, AfiMI0165UVic, AfiMI0196UVic, AfiMI0240UVic, AfiMI0304UVic, AfiMI0411UVic, AfiMI0416UVic).

The 24 linkage groups span 1332.8 cM in the merged map, with individual linkage groups ranging from 20.9 cM (LG22) to 80.3 cM (LG15). A significant difference in recombination was observed between the sexes as the male map had a length of 860.4 cM, while the female map had a length of 1610 cM; this gives a female:male recombination rate of 1.87:1, although the recombination rate in individual linkage groups varied greatly. This is not unusual, as higher recombination rates have been reported in the females of a number of fish species, including Atlantic salmon [14], catfish [15], rainbow trout [40] and zebrafish [41]. In contrast, the recombination rates between the individual fathers or between the individual mothers of our mapping families were both approximately 1:1. The overall length and female/male recombination ratios are likely to be underestimates however, as there are still a few gaps in the female-specific linkage map.

With only 2 markers remaining unlinked after mapping, we were confident that all of the chromosomes were covered by the map. This was subsequently confirmed after publication of the sablefish karyotype [42]; a diploid chromosome number of 2n = 48 was identified, the same number as was predicted here through linkage mapping. Of note, this karyotype represents the most-commonly identified karyotype in teleost fish, and is thought to represent that of the common teleost ancestor after the last whole-genome duplication [43].

### Significant conserved synteny observed between *A. fimbria* and *G. aculeatus*

A comparison to the most closely related species for which a whole genome sequence is available was undertaken to look for syntenic chromosomes and conservation of gene order. All of the markers were derived from EST assembled transcripts. These EST contigs, which include markers that mapped to one of the 24 linkage groups, or the 2 singletons, were BLATed against the threespine stickleback genome. Most (278/360) of these contig comparisons produced a significant “hit” to the stickleback genome; furthermore, as can be seen in Table 1, the level of conserved synteny between the two species is quite high, with a large number of markers from one sablefish linkage group producing significant “hits” to the corresponding stickleback chromosome. In most cases, the relationship between stickleback and sablefish is 1:1 – that is, one stickleback chromosome corresponds to a single sablefish linkage group. In three cases, there were two linkage groups for one stickleback chromosome. Analyses of end-markers, however, show no hints of linkage between any of the three pairs of linkage groups. This is not surprising given the haploid chromosome number in the threespine stickleback is 21 while the haploid karyotype for sablefish is 24. We used these relationships to assign numbers to each of our linkage groups to make comparison to stickleback easier; thus LG02 corresponds to stickleback ChrII, and LG11 corresponds to stickleback ChrXI. In the case of the three linkage group pairs BLATing to single stickleback chromosomes, the largest linkage group was assigned the stickleback chromosome number, while the smaller received the next number available above 21; resulting pairs were LG01 and LG22, LG04 and LG23 and finally LG07 and LG24.

**Table 1:** Comparative synteny between threespine stickleback and sablefish showing the number of sablefish marker loci with significant BLAT hits to the stickleback genome and the predicted orthologous chromosomes for each linkage group

									**Sablefish linkage groups**																
		1	2	3	4	5	6	7	8	9	10	11	12	13	14	15	16	17	18	19	20	21	22	23	24
	**I**	**6**	**-**	**-**	**-**	**-**	**-**	**-**	**-**	**-**	**-**	**-**	**-**	**-**	**-**	**-**	**-**	**-**	**-**	**-**	**-**	**-**	**3**	**1**	**-**
	**II**	-	**10**	**-**	**-**	**-**	**-**	**-**	**-**	**-**	**-**	**-**	**-**	**-**	**-**	**-**	**-**	**-**	**-**	**-**	**-**	**-**	**-**	**-**	**-**
	**III**	-	-	**16**	**-**	**-**	**-**	**-**	**-**	**-**	**-**	**-**	**-**	**-**	**-**	**-**	**-**	**-**	**-**	**-**	**-**	**-**	**-**	**-**	**-**
	**IV**	-	-	-	**14**	**-**	**-**	**-**	**-**	**-**	**-**	**-**	**-**	**-**	**-**	**-**	**-**	**-**	**-**	**-**	**-**	**-**	**-**	**5**	**-**
	**V**	-	-	-	-	**14**	**-**	**-**	**-**	**-**	**-**	**-**	**-**	**-**	**-**	**-**	**-**	**-**	**-**	**-**	**1**	**-**	**-**	**-**	**-**
	**VI**	-	-	-	-	-	**8**	**-**	**-**	**-**	**-**	**-**	**-**	**-**	**-**	**-**	**-**	**-**	**-**	**-**	**-**	**-**	**-**	**-**	**-**
	**VII**	-	-	-	-	-	-	**13**	**-**	**-**	**-**	**1**	**-**	**-**	**-**	**-**	**-**	**-**	**-**	**-**	**-**	**-**	**-**	**-**	**3**
	**VIII**	-	-	-	-	-	-	-	**13**	**-**	**-**	**-**	**-**	**-**	**-**	**-**	**-**	**-**	**-**	**-**	**2**	**-**	**-**	**-**	**-**
	**IX**	-	-	-	-	-	-	-	-	**14**	**-**	**-**	**-**	**-**	**1**	**-**	**-**	**1**	**-**	**-**	**-**	**-**	**-**	**-**	**-**
	**X**	-	-	-	-	-	-	-	-	-	**12**	**-**	**-**	**-**	**-**	**-**	**-**	**-**	**-**	**-**	**-**	**-**	**-**	**-**	**-**
**Stickleback chromosomes**	**XI**	-	-	-	-	-	-	-	-	-	-	**20**	**-**	**-**	**-**	**-**	**-**	**-**	**-**	**-**	**-**	**-**	**-**	**-**	**-**
	**XII**	-	-	-	-	-	-	-	-	-	-	-	**19**	**-**	**-**	**-**	**-**	**-**	**-**	**-**	**-**	**-**	**-**	**-**	**-**
	**XIII**	-	-	-	-	-	-	-	-	-	-	-	-	**8**	**-**	**-**	**-**	**-**	**-**	**-**	**-**	**-**	**-**	**-**	**-**
	**XIV**	-	-	-	-	-	-	-	-	-	-	-	-	-	**15**	**-**	**-**	**-**	**-**	**-**	**1**	**-**	**-**	**-**	**-**
	**XV**	-	-	-	-	-	-	-	-	-	-	-	-	-	-	**9**	**-**	**-**	**-**	**-**	**-**	**-**	**-**	**-**	**-**
	**XVI**	-	-	-	-	-	-	-	-	-	-	-	-	-	-	-	**14**	**-**	**-**	**-**	**-**	**-**	**-**	**-**	**-**
	**XVII**	-	-	-	-	-	-	-	-	-	-	-	-	-	-	-	-	**11**	**-**	**-**	**-**	**-**	**-**	**-**	**1**
	**XVIII**	-	-	-	-	-	-	-	-	-	-	-	-	-	-	-	-	-	**14**	**-**	**-**	**-**	**-**	**-**	**-**
	**XIX**	-	-	-	-	-	-	-	-	-	-	-	-	-	-	-	-	-	-	**7**	**-**	**-**	**-**	**-**	**-**
	**XX**	-	-	-	-	-	-	-	-	-	-	-	-	-	-	-	-	-	-	-	**14**	**-**	**-**	**-**	**-**
	**XXI**	-	-	-	-	-	-	-	-	-	-	**1**	-	-	-	-	-	-	-	-	-	**8**	**-**	**-**	**-**
	**Un**	-	-	**-**	-	-	**1**	-	**1**	-	**2**	**1**	**2**	**3**	**1**	**1**	**-**	**7**	-	-	-	**2**	**-**	**1**	**2**
**Markers with no significant hits**		**0**	**1**	**2**	**0**	**2**	**2**	**0**	**6**	**4**	**1**	**3**	**5**	**1**	**1**	**2**	**3**	**2**	**4**	**7**	**2**	**2**	**2**	**2**	**2**
**Total number of markers**		**6**	**11**	**18**	**14**	**16**	**11**	**13**	**20**	**18**	**15**	**26**	**26**	**12**	**18**	**12**	**17**	**21**	**18**	**14**	**20**	**12**	**5**	**9**	**8**
**Orthologous to stickleback chr:**		**I**	**II**	**III**	**IV**	**V**	**VI**	**VII**	**VIII**	**IX**	**X**	**XI**	**XII**	**XIII**	**XIV**	**XV**	**XVI**	**XVII**	**XVIII**	**XIX**	**XX**	**XXI**	**I**	**IV**	**VII**

Linkage groups showed anywhere from 3 (LG24) to 17 (LG04) hits to a single stickleback chromosome. Only 8 of the 278 loci that were located to the 21 chromosomes in the stickleback produced significant hits to chromosomes other than that predicted for the linkage group. Six of these eight markers were located 3 markers or less from the end of the linkage group. An additional 24 loci, interspersed throughout the linkage groups, hit the stickleback chrUn, a “chromosome” composed of all sequence data unassigned to one of the 21 chromosomes. While most linkage groups had 0–2 loci that hit this “Un” chromosome, 7 hits were from loci assigned to linkage group 1. Only one of the two un-linked microsatellite markers had a positive BLAT hit, mapping to ChrI in the threespine stickleback; this suggests that the chromosome represented by either LG01 or LG22, the two linkage groups that are predominately associated with stickleback ChrI, is likely larger than predicted by the current sablefish linkage map.

**Figure 2:**
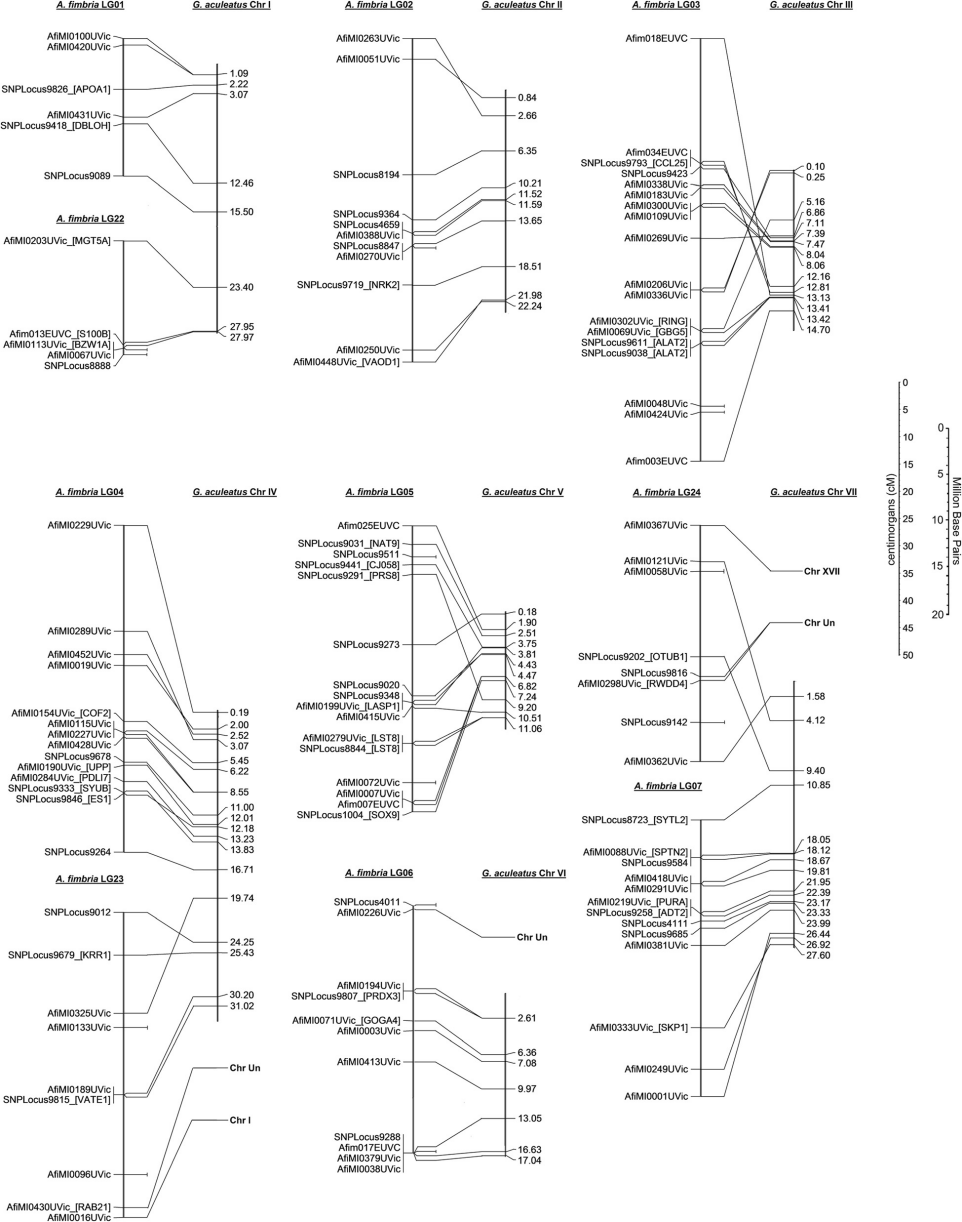
**Conserved synteny between sablefish and threespine stickleback stickleback Chromosomes I-VII.** A line is drawn to compare the position of a marker on a linkage group to the strongest BLAT hit (>100 BLAT score) for the contig sequence used in primer design.

**Figure 3:**
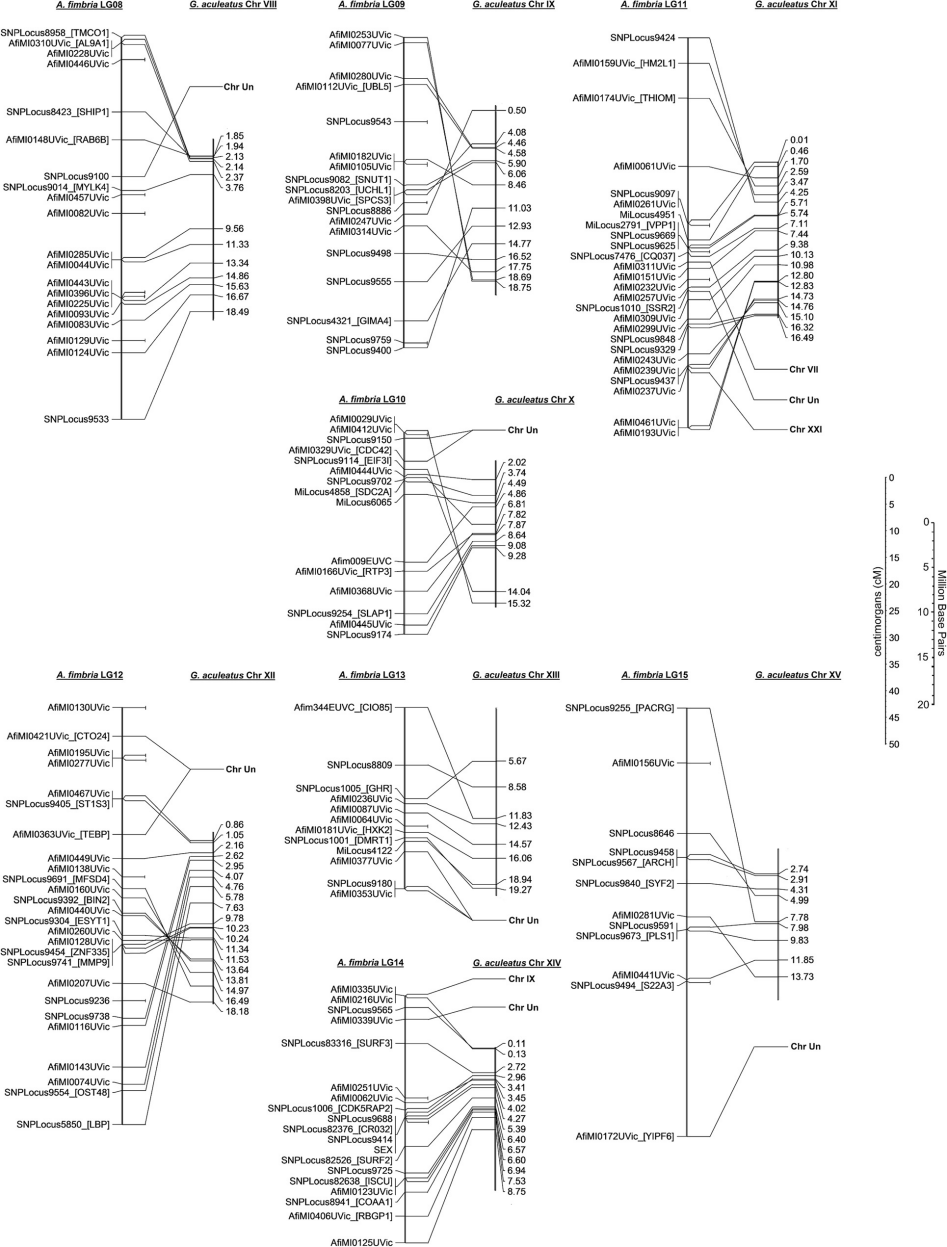
**Conserved synteny between sablefish and threespine stickleback stickleback Chromosomes VIII-XV.** A line is drawn to compare the position of a marker on a linkage group to the strongest BLAT hit (>100 BLAT score) for the contig sequence used in primer design.

**Figure 4:**
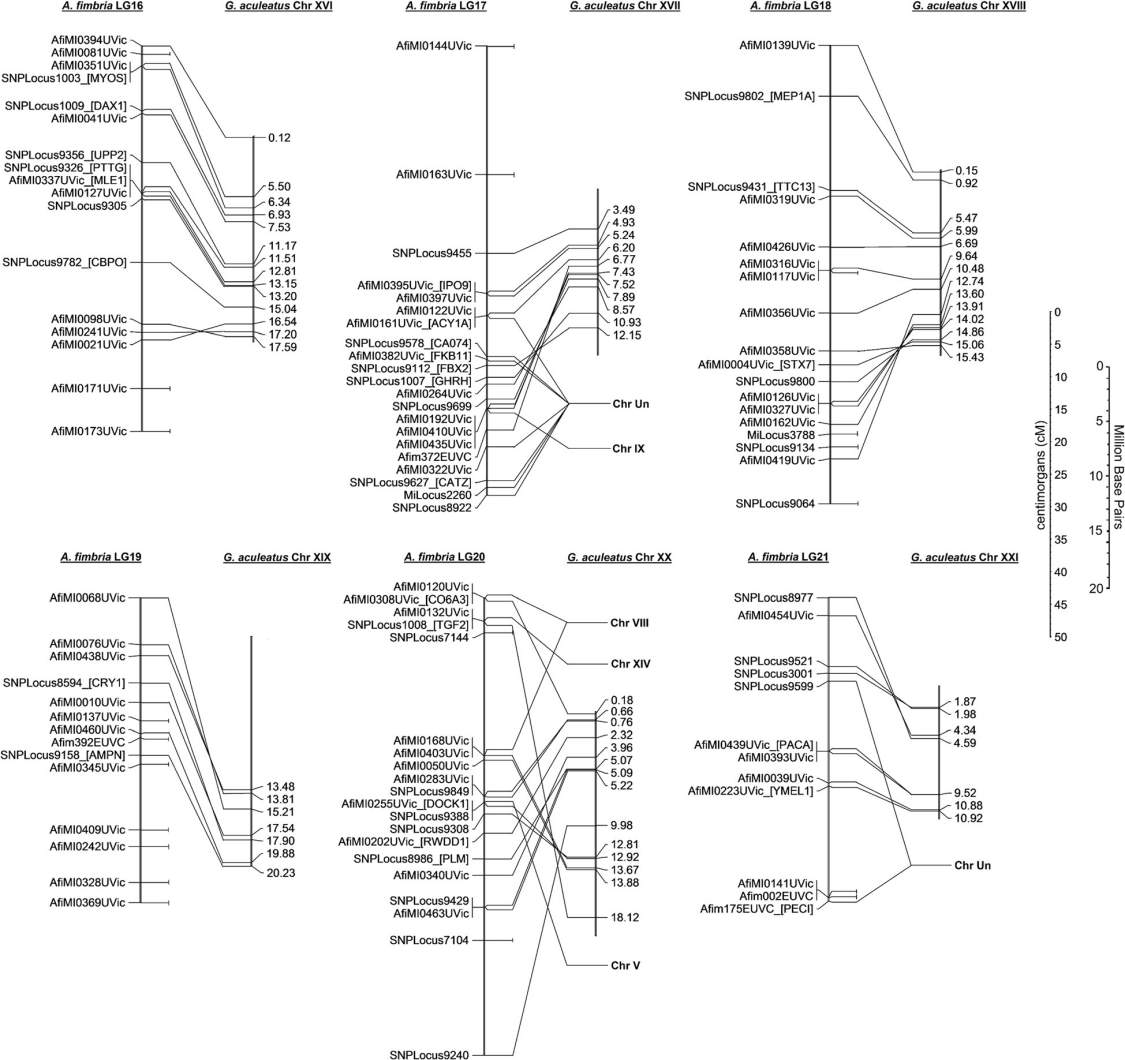
**Conserved synteny between sablefish and threespine stickleback stickleback Chromosomes XVI-XXI.** A line is drawn to compare the position of a marker on a linkage group to the strongest BLAT hit (>100 BLAT score) for the contig sequence used in primer design.

Gene order also appears relatively well conserved between sablefish and the threespine stickleback, as shown in Figures 2, 3, 4. In most cases, 1–3 inversions can be used to explain the difference in marker ordering between the two species, with markers located between inversion points consistently ordered. This is important and will be revisited later, as it allows for prediction of candidate genes of interest in the sablefish based on the annotated whole-genome sequence in stickleback. Development of the sablefish linkage map and the identification of conserved synteny may present an opportunity to re-evaluate the stickleback genome assembly and successfully locate some of the loci previously pooled into chr Un.

### Additional markers by genotyping-by-sequencing (GBS)

The construction of a GBS library was performed to create a second-generation linkage map to supplement our microsatellite/SNP sablefish map and to identify potential sex-specific sequences. Two Illumina lanes of GBS sequencing were produced to test this technique for sablefish linkage mapping and to determine whether the *Ape*KI restriction enzyme used was appropriate for sablefish; most of the individuals were from our second mapping family, with a few additional, unrelated and sexed individuals used to aid in sex identification (see following section). We obtained ~490 million barcoded reads, with between 500,000 and 1,300,000 unique reads per individual. We determined there was an average of 8–10 fold read coverage per unique genomic sequence, well below the desired 60x for a *de novo* sequencing study [44]. While overall coverage was less than desired, we were still able to utilize the data to identify additional SNP markers. With relatively conservative parameters, a total of 1843 polymorphic loci were identified through next-generation sequencing; see Additional file 6: Table S4 for details.

### Genetic identification of SEX in sablefish

In sablefish, as in other fish species, one of the most economically and biologically important and perplexing phenotypes is sex. Sexual dimorphism in fish is often observed with one sex, commonly the female in sablefish, growing both faster and larger than the other sex. Rapid and substantial growth is a desirable trait particularly in aquaculture and ocean ranching. Thus, selection for females early in rearing, or the production of monosex female offspring is of great interest to these industries. While external identification of sex is difficult and inaccurate in immature sablefish, internal analysis of the gonads can usually determine the sex of the fish but sacrificing the fish is required. At 15 months, the sex of each of our fish was easily identified by the size of the gonads themselves, with the ovaries well over twice the size of the testis in males and females of equal size and weight. Assuming a single locus phenotype, we attempted to place sex on each of the sex-specific maps. While unsuccessful in linking sex to the female map, we were successfully able to map the trait to linkage group 14 of the male map, suggesting an XX-XY sex determination system. As previously described in the comparative mapping section, this corresponds to chromosome XIV of the stickleback genome. Adjacent markers further narrowed this region to between 3.0 and 5.4Mb on the stickleback genome. This region does not correspond to the Y-specific region of the threespine stickleback linked to Chr XIX [45] nor to the Y-chromosome associated with LG12 (Chr XII) of the ninespine stickleback *Pungitius pungitius*[46]. During review, a report was published on the expression of five genes of interest in sex, including three shown to be significantly elevated in juvenile testes [47]. Two of these three are located on the linkage map, *dmrt1* mapping to LG13 and *sox9a* to LG05, both predicted by comparative mapping to the threespine stickleback genome. Neither the remaining testis-elevated gene, *amh*, nor the two genes found with elevated expression in the ovaries, *foxl2* and *cyp19a1a* were predicted to be found on LG14; *amh* is predicted to be located on LG08, *foxl2* on LG01 and *cyp19a1a* on LG02.

Examination of the sablefish GBS library was undertaken to determine whether we could identify any sex-specific sequences. The sequences were processed using the program Jellyfish [48] into kmers with length 31 and each kmer was counted in each individual, and placed into a matrix. We searched for unique kmers found only in male or female fish, with a minor allowance for sequencing errors (max two individuals). After assembling sex-specific kmers into overlapping mini-contigs, we were able to identify 11 mini-contigs of interest, which were then mapped back to the original 100 bp paired-end reads. These reads were BLATed against the stickleback genome, and two of these reads produced hits >50 (default BLAT score), both of which BLATed against the region where we expected to find sex in ChrXIV of stickleback. These sites have been designated Tag2 and Tag10 based on the order that they were identified. Genome walking was performed in order to provide enough sequence to design primers to score the polymorphic markers in our sex-specific tags, and this process recovered well over 1000 bp surrounding each tag for primer design. Following amplification with the new primers on 53 unrelated, definitively sexed sablefish, the alleles identified by Tag 2 and Tag10 were present in every male and absent in all females.

Both tags BLAT to the region between the genes PPEF2 and AFF1 on the stickleback genome. In this region, and in particular between these two tags, two coding regions were predicted to be found based on stickleback ESTs. Based on tBLASTx, the first coding region (ESTs GenBank: DW615685, DN718296, DW662322, DN733719, DT981615) was an *aff1* homolog. The second gene region (ESTs GenBank: CD507187, DW624794) was most likely gonadal soma-derived factor (*gsdf*). Primers were designed to amplify the region between our tags and a short region of *gsdf* available in our sablefish RNAseq assembly. Amplification of the long PCR products that stretched between Tag10 and gsdf revealed a male specific fragment that was ~ 500 bp larger than the PCR products obtained from female samples. No differences between male and female samples were observed between the coding region of *gsdf* and Tag 2 (data not shown).

**Figure 5:**
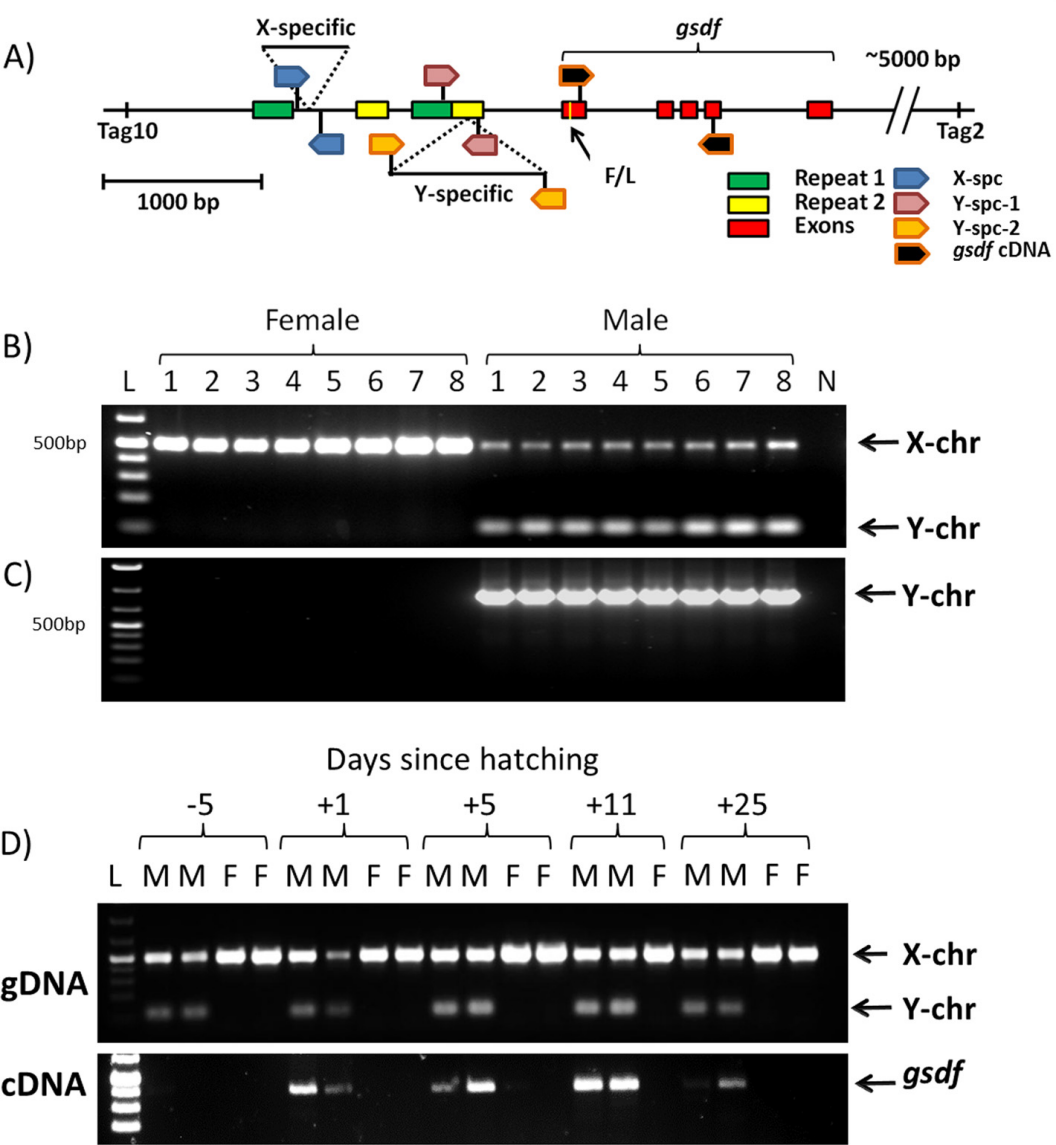
**Sex-specific sequences in sablefish. A)** The major features of the sablefish sex region, including the relative location of the X and Y-specific insertions to the start of the *gsdf* coding region and the location of the subsequent PCR reaction primers **B)** PCR reaction showing the amplification of a single, larger fragment in 8 unrelated female fish, and two fragments in the males with the Y-fragment 412bp smaller in size **C)** Nested PCR demonstrating the presence of the Y-specific insertion solely in the males. **D)** time-series of pre-hatch and post-hatch sablefish showing genetic sex-identification based on the X-specific insertion, and *gsdf* expression in these individuals. For **B)**, **C)** and **D)** “L” represent the 1kb o’generuler plus ladder (Thermo Scientific), with the brightest two bands at 500 and 1500bp, “N” represents the negative control.

Primers were designed to span the region between Tag 10 and *gsdf* in order to identify the male-specific sequences. Sequencing the resulting amplified products yielded a number of interesting features in the sequence upstream and within the *gsdf* gene (see Figure 5A). Two regions of ~180 bp and ~140 bp are each present in duplicate (95% identity) in the sequence 5’ of the gene, while a number of smaller repeats are found throughout the sequence. A number of SNPs in the upstream and intronic portions of *gsdf* appeared to be linked to sex and there were two exonic polymorphisms, one of which caused a sex-specific coding change from a phenylalanine in the X-chromosomal copy to a leucine in the Y. Most surprising though was the presence not only of a Y-specific sequence of 935 bp, but an X-specific sequence of 412 bp that produced the observed 500 bp difference in amplified size. Primers were designed to confirm X and Y specificity of the sequences. A PCR reaction was designed to amplify across the X-specific sequence, with primers designed just outside of the specific sequence to produce amplified bands from both chromosomes; as seen in Figure 5B, amplification in both sexes show the larger band corresponding to the X-chromosome, while only amplification in the males produced the small band from the Y-chromosome demonstrating the lack of X-specific sequence. Amplification of the Y-specific sequence was more difficult due to the insertion falling in the middle of a repeated sequence; a nested PCR was therefore performed targeting only the male-specific sequence. As shown in Figure 5C, the nested primers produced bands only in the males, not in the females. In the 53 unrelated, definitively sexed sablefish analyzed, all tested females carried two copies of the X-specific insertion, while males carried one chromosome with the X-specific insertion and one with the Y-specific, adding further evidence to the male being the heterogametic sex. Both sequences are flanked by inverted repeats of 12 (Y-chr) to 16 (X-chr) base pairs, suggesting the original insertion may have involved a transposase, although no transposase could be identified in the inserted sequences. Representative sequences for the X and Y insertions were uploaded to the NCBI nucleotide database as Genbank:KC623942 and Genbank:KC623943 respectively; masked sequences with all described elements can be found in Additional file 7: Figure S3.

Portions of the X-specific sequence were identified elsewhere in the sablefish TSA library, but with no strong hits to the adjacent *gsdf* promoter sequences it is likely that the 412 bp sequence is a repeat sequence or transposable element. Significant BLAST hits for this element to non-sablefish sequences were to EST and WGS sequences of Atlantic cod, *Gadus morhua*, (91-100% max identity). The Y-specific insertion also only produced significant BLAST hits to *G. morhua* sequences, although the Y-specific insertion was not observed in the expressed sablefish sequences. Even though sablefish is commonly called Alaskan black cod, it is not a true cod and is only distantly related to the Gadiformes (last common ancestor ~130-200mya [38,39]). While the mechanism for the observed sequence similarity between this element in the two species remains unknown, others have proposed that parasites such as the trematodes *Schistosoma japonicum* and *Diplostomum* spp. [49], or the sea lamprey *Petromyzon marinus*[50] may facilitate lateral transposition of transposable elements and other sequences between unrelated host fish species; Atlantic cod and sablefish are geographically separated but the range of the Pacific cod *Gadus macrocephalus* and sablefish overlap significantly allowing for the possibility of some form of lateral transfer between species.

The presence of the major sex-specific insertions in sablefish immediately upstream of the *gsdf* gene is quite intriguing. As a member of the transforming growth factor-β superfamily, the *gsdf* gene was first described in the proliferation of primordial germ cell and spermatogonial proliferation in rainbow trout [51]. It has since been shown that in the evolutionarily conserved cluster of genes that includes *gsdf*, it is the only gene preferentially expressed in teleostean testis [52]. Further, it has been shown that expression of *gsdf* in the zebrafish occurs prior to gonad differentiation in the early development of zebrafish, and is expressed solely in the gonads [52]. The gene was described as a master-sex determinant in a medaka species, *Oryzias luzonensis*[27]. While we do not present enough evidence to describe *gsdf* as the master-sex determinant in sablefish, the location of the sex-specific insertions (or deletions) in the promoter region immediately upstream of a gene that has been described as a master sex determinant in another fish species, and coupled with its placement in a cluster of genes otherwise expressed preferentially in ovaries suggests that *gsdf* is the strongest candidate for the master-sex determinant in sablefish. Furthermore, if future research supports gsdf as the master-sex determinant in sablefish, it will represent the first description of the independent evolution of the same gene to the role of master-sex determinant in different orders of fish. Independent evolution of DMRT as a sex-determinant has been described in African clawed frogs [53] and chickens [54], but in fish, this gene has been described as the main sex-determinant only in closely related medaka species.

From *Oryzias luzonensis*, it is estimated that sex-specific expression differences in *gsdf* are first observed around hatching (10 days post fertilization [d.p.f]), and are no longer detected at 10 days after hatching [27]. If the same timing was observed in sablefish, significant male over-expression should be observed between around 15.5 d.p.f (4°C incubation) [55] and will no longer be significant 15 days post-hatching (d.p.h). As shown in Figure 5D, *gsdf* is overexpressed in males around hatching, although unlike our predictions, it remains overexpressed relative to the females up to 25 d.p.h.; products are not visible following 35 cycles in either sex five days prior to hatching. In 15 month sablefish, no difference is observed in the expression of *gsdf* in ovaries and testis (data not shown). It is therefore possible that differential expression may contribute to the development of each sex, although it remains to be determined what role the amino acid substitution from phenylalanine to leucine may have on the actions of the protein.

### Significance of the genomic tools

Genomic tools can be applied to many questions relating to fisheries, aquaculture, aquatic physiology, ecology, evolution, disease, resistance, reproduction, growth, response to the environment, general immunity and general biology of sablefish. The resources developed here will be fundamental in the management and analysis of wild and domestic commercial stocks. At a landed value of US $4.46/lb, the sablefish is the second most valuable finfish per pound (after halibut, US $4.97) and represents the fourth greatest total value (after walleye pollock, sockeye salmon, and Pacific halibut, ~equal to Pacific cod) to 2011 Pacific Canadian and American commercial fisheries [5,6]. The large set of microsatellite and SNP markers as well as the sequences provided by the complete mitochondrial genome, will allow for a robust molecular analysis of sablefish structure throughout the natural range to determine the most appropriate population management. Additionally, the ease of genetically sexing both immature and mature sablefish will allow for the easy addition of sex as an additional parameter in analyses of population composition and movement. The availability of expressed sequences should allow a genetic base to design studies to gain insight into the physiology and biology of these deep-sea organisms.

Many of these markers can likely be used in analysis of closely related species such as Skilfish (*Erilepis zonifer*) [9] and perhaps other Scorpaeniformes, Gasterosteiformes and Perciformes species. The degree of conserved synteny between the threespine stickleback and the sablefish suggests that these tools may be very useful in many species including other economically important species such as lingcod and greenling (Family: Hexagrammidae) or the more distantly related rockfish (Family: Sebastidae).

The use of these resources will also be helpful in the development of species for aquaculture as well as for conservation and management of wild stocks. A linkage map and the demonstrated ability to use the threespine stickleback genome to determine likely gene location will facilitate molecular analysis and development of markers for selective breeding. The expressed transcript library will provide access to particular genes of interest and the development of sex markers will allow for the study of sex-specific phenotypes. If *gsdf* is confirmed as the master sex determinant in sablefish, the targeted production of monosex or sterile stocks for use in aquaculture may be possible.

## Conclusions

With this work, we present the largest collection of genetic data available for sablefish. A mitochondrial genome sequence was produced and used to place sablefish among Scorpaeniformes and Gasterosteiformes fishes. We have sequenced and assembled transcript library of nearly 35,000 sequences for the sablefish, and identified microsatellite and SNP markers for use in broodstock selection and population management. Using our polymorphic markers, we have produced a linkage map consisting of 24 linkage groups, which is in agreement with the expected number of chromosomes previously identified through karyotyping. Comparative mapping has been used with *G. aculeatus* to show significant conservation of gene order, allowing for the stickleback genome to be used to predict gene location in sablefish. A genotyping-by-sequencing library was used to identify additional SNPs, adding 1843 markers, and to identify sex-specific markers. Finally, the sex-specific markers led directly to the identification of sex-specific sequences in the sablefish, located in the upstream promoter region of the known sex pathway gene, *gonadal soma-derived factor*, the master sex determinant in the medaka, *Oryzias luzonensis*.

## Methods

### Sample Collection and extractions

All sablefish used in this work were provided by Sablefish Canada Ltd. The first family is the result of a paired hatchery mating, with a single father and mother and 83 progeny. Tissue collection from each parent was through fin clip, while the samples from the progeny are through whole body DNA extractions on samples collected just after hatching, all stored in 95% ethanol until use. The second family, also a result of hatchery mating, produced a half-sibling family from three males and a single female. For each of the parents and the 115 progeny, fin clips were used as source of DNA (stored in 95% ethanol). The sex of 100 fish in the second family was recorded by the appearance and size of the gonads at 15 months of age and liver, spleen, kidney, gonad and head kidney tissues were collected, initially frozen on dry ice, and stored long-term at −80°C. DNA was isolated using a Chelex extraction protocol following the protocol of [56]. A phenol DNA extraction was performed on the liver tissue of one of the progeny from family 2 following a standard protocol [57]; this extraction was used for mitochondrial genome sequencing. Liver tissues from family 2 progeny and fin tissue from the parents and 14 immature, unrelated sablefish (8 males, 6 females) were extracted for use in Genotyping-by-Sequencing using the standard protocol in the DNeasy Blood & Tissue Kit (Qiagen). For sex-specific sequencing, fin clips from 47 mature, definitely sexed broodstock were extracted by Chelex extraction and used along with the 6 family parents.

Total RNAs were extracted from each tissue in TRIzol reagent (Invitrogen) by mixer-mill homogenization (Retsch) and spin-column purified using RNeasy Mini kits (Qiagen). Each RNA sample was then quantified and quality-checked by spectrophotometer (NanoDrop Technologies) and agarose gel, respectively. For cDNA libraries, the brain, gill and kidney were taken from sablefish unrelated to the mapping families and processed. For RNAseq, RNA was extracted from the kidney, liver and gonadal tissues from one male and one female sablefish from the second mapping family.

For time-series samples, sablefish were collected 5 days before and 1, 5, 11 and 25 day after hatching, and placed in RNAlater (Invitrogen) until use. Whole larvae were digested with Proteinase K (Qiagen); following digestion, 25% of the sample was used with the DNeasy Blood & Tissue Kit (Qiagen) while the remaining sample was purified using the RNeasy mini kit (Qiagen). RNA samples were converted to cDNA for PCR using M-MuLV Reverse Transcriptase (NEB).

### cDNA libraries

*Anoplopoma fimbria* EST libraries were constructed using the methods of Koop et al. [58]. In short, the library was constructed directionally in pAL17.3 (Evrogen Co.). Libraries were plated and colonies picked using a Qpix2 array picker. Following overnight growth of glycerol stocks arrayed in 384-well format [59], plasmid DNA was extracted using a standard lysis and neutralization procedure followed by an alcohol precipitation. Sequencing was performed on an ABI 3730 sequencer using BigDye™ Terminator V3.1 (ABI) cycle sequencing kit and either the primer M13 forward (5’-GTAAAACGACGGCCAGT-3’) for 5’ end or SP6WAN (5’-ATTTAGGTGACACTATAG-3’) for 3’ end sequencing. Base calling from traces and quality scores assigned using Phred [60,61], and Phrap used to assemble the sequences into contigs (200 minscore, 0.99 stringency; http://www.phrap.org/).

One lane of RNAseq 100 bp PET Illumina sequencing was run on a HiSeq2000 sequencer, and assembled using SOAPdenovo (k=75) [62]; sequencing was performed at the Beijing Genomics Institute.

RNAseq and EST library data were merged into a Transcriptome Shotgun Assembly. Contigs larger than 200 bp from both datasets were assembled using Phrap (200 minscore, 0.96 stringency), with resulting contigs 400 bp or larger retained. Contigs were annotated using SwissProt and Gene Ontology’s annotated protein databases using a threshold of 1e-5.

### Mitochondrial genome sequencing

A slightly modified version of Miya and Nishida’s protocol [63] was used to amplify the mitochondrial genome in two long, overlapping sequences with smaller, secondary PCRs for sequencing. Primers were designed to the 16S ribosomal RNA and cytochrome B mitochondrial sequences in NCBI (accessions GU018112 and FJ264496 respectively). Afim-Cb-H (5’-GATATGAGCCGTAGTAAAGACCTCGGCCGA-3’) and Afim-16S-L (5’-TCGACAAGGGGGTTTACGACCTCGATGTTG-3’) were designed to the same place as the Gogr-Cb-H and Gogr-16S-L primers and these primers were used to amplify the larger mitochondrial fragment, covering the NADH dehydrogenase, cytochrome oxidase and ATPase genes. Afim-16S-H (5’-GACCTGGATTACTCCGGTCTGAACTCAGAT-3’) was also designed to the same place as Gogr-16S-H; however, only the 3’ end of the primer was found in the available sequence, and the 5’ end of the designed primer comes directly from Gogr-16S-H. Afim-Cb-L (5’-GATTAATCCGAAACATTCACGCTAACGGTG-3’) was designed to a different portion of the Cytochrome B sequence, as Gogr-Cb-L did not align to the available Cytochrome B sequence. These two primers were used to amplify the small mitochondrial fragment, which contained the 12S ribosomal RNA and the D-loop. Both reactions were amplified with the Phire Hot Start II DNA polymerase (Finnzymes); 1× Phire reaction buffer, 0.2 µM of each dNTP (Promega), 0.5 µM of each primer (IDT), 50 ng genomic DNA and 0.5 µl enzyme in 25 ul reactions. Reactions were loaded onto a Techne TC-412 at 98°C for 45 seconds, followed by 35 cycles of 98°C for 8 seconds, 63°C for 10 seconds and 72°C for 4 minutes. Following a final extension of 72°C for 8 minutes, samples were cooled to 4°C. After confirming successful amplification on a 1% agarose gel, the reactions were diluted 1/100, and used as templates for the secondary PCRs. The 30 pairs of fish-universal primers [63] and the four long primers were used to amplify small pieces of the mitochondrial genome in an overlapping fashion; when a pair could not successfully amplify a product (ie. 5-L/5-H), an adjacent primer was used instead (ie. 5-L/6-H); long primers were used when overlap between the two large fragments could not be obtained. 25 µl reactions were used, containing 1× GoTaq Flexi Buffer (Promega), 2.5 mM MgCl2 (Promega), 0.2 µM each dNTP (Promega), 0.5 µM each primer (IDT) and 0.625U Hot Start GoTaq polymerase (Promega). Samples were amplified on Techne TC-412 at 95°C for 3 minutes, followed by 30 cycles of 95°C for 15 seconds, 45°C for 15 seconds and 72°C for 45 seconds, with a final extension of 10 minutes at 72°C, and a final hold of 4°C. The PCR products were plate purified with Qiagen MinElute 96UF PCR purification; samples were eluted in 20 µl DNAse/RNAse free water. Sequencing reactions were prepared containing 0.5 μl BigDye Terminator v3.1 (ABI), 0.5 µl BigDye Terminator sequencing buffer, 0.64 μM forward or reverse primer and approximately 20 ng purified PCR product in a 5 ul reaction; these were run on a TC-412 (Techne) as follows: 1 min 95°C initial denaturation, followed by 30 cycles of 95°C for 30 s, 50°C for 15 s, and 60°C for 90 s and a final extension of 5 min at 72°C. Sequencing reactions were ethanol precipitated, and re-suspended in 20 μl DNAse/RNAse-free H_2_O (Gibco). All sequencing was performed on an ABI 3730 DNA analyzer. Resulting sequences were assembled using the assembler in Geneious v5.1.7. Mitochondrial features were identified using DOGMA [64] and by comparison to the available mitochondrial genomes of other Scorpaeniformes: *Aptocyclus ventricosus* (Genbank:AP004443), *Clinocottus analis* (Genbank:FJ848374), *Cottus hangiongensis* (Genbank:EU332751), *Cottus poecilopus* (Genbank:EU332750), *Cottus reinii* (Genbank:AP004442), *Helicolenus hilgendorfii* (Genbank:AP002948), *Satyrichthys amiscus* (Genbank:AP004441), *Sebastes schlegeli* (Genbank:AY491978), *Sebastiscus marmoratus* (Genbank:GU452728).

For phylogenetic analysis, the RAXML (7.0.4) protocol of Kawahara et al. [35] was followed. Along with 71 of the 75 mitogenomes previously analyzed, we added 15 mitogenomes more recently uploaded to NCBI that were expected to be closely related to the Scorpaeniformes (see Additional file 8: Table S5 for the list), as well as the sablefish mitogenome. Following the trimming of ambiguously aligned sections, gaps and loop structures, we focused on 13528 bases: 10436 in the 12 protein-coding genes (excludes ND6, see [35]), 1411 bases in the 22 tRNAs, and 1681 in the two rRNA sequences. For the 12 protein-coding genes, we re-assigned the third position in each codon as either purine (R) or pyrimidine (Y) as deemed the best estimate and conservation of signal to noise [35].

### Primer development and marker identification - SNPs

SNP primer design was performed with three approaches. For the first batch, assembled contigs were analyzed for variations in individual EST sequences at single bases. Using primer3 [65], primers amplifying products of 200-400 bp containing the expected SNP were designed, with primers of 18–22 bp with T_m_≈ 55°C, max 5°C difference in T_m_ and a 2 bp GC clamp. In the second batch, primers were designed to a random selection of EST contigs, with a focus on the 3’ UTR. Again using primer3, primers amplifying larger products, 400-550 bp, were designed, with the same characteristics as above. Finally, one set of primers was designed to target each of *dmrt1, cdk5rap2*, myostatin, growth hormone receptor, growth hormone receptor hormone, *ssr2, tgf2, dax1 and sox9* loci. Alignment of the loci from the species *Gasterosteus aculeatus*,* Oryzias latipes, Takifugu rubripes*, and *Tetraodon nigroviridis* were visualized using the UCSC genome browser and primers were designed to highly conserved sequence in the four species. PCR and sequencing reactions were performed using the SNP amplification and sequencing protocol in Messmer et al. [56]; all sequences were obtained using an ABI 3730 DNA analyzer. Sequences from each primer pair were aligned in Geneious 5.1.7 and SNPs identified and scored manually.

### Primer development and marker identification – microsatellites

Using the program RepeatFinder [66], repeats of 2–5 nucleotides length (min 4 repeats) were identified from the assembled EST transcripts. Using primer3, primers were designed to amplify products 75 – 200bp in size encompassing the expected repeat, with primers of 18–22 bp with T_m_≈55°C and max 5°C difference in T_m_. An additional 13 primer pairs designed from the same dataset were used, and are described in [8].

PCR reactions and labelled microsatellite genotyping followed the microsatellite amplification and scoring protocol in Messmer et al. [56]. In initial primer testing, successful amplification and polymorphism, were determined using 10% polyacrylamide gels (25 ml of 40% 19:1 acrylamide: bis-acrylamide (BioRad); 100 ul TEMED (Sigma); 1ml 10% w/v ammonium persulphate (Sigma); 74 ml H_2_O) run for 12–14 hours, stained using EtBr and imaged; half the microsatellites in family 1 were also analyzed through this method. The remaining microsatellites scored in family 1 and all microsatellites scored in family 2 included either a 6-FAM or HEX labelled fluorescent dye (IDT) attached to one of the primers. Following amplification, 0.5 µl each PCR product (one HEX-labelled and one 6-FAM labelled) was added to 9.9 µl Hi-Di™ Formamide (ABI) and 0.1 μl GeneScan™ -500 ROX™ Size Standard (ABI), and samples denatured by heating to 95°C for 3 min and placed on ice for 5min. Amplifications were run on a ABI 3730 DNA sequencer and electropherograms were analyzed using GeneMapper V4.0 (ABI).

### Marker naming

Nomenclature for newly developed markers follows a modified version of the microsatellite nomenclature of Jackson et al. [67]. The species was identified by the first letter of the genus followed by 2 of the species (Afi); marker type was represented by a two letter identifier (MI = microsatellite, SP = SNP); a unique four digit number was assigned to each of the individual markers; a four letter identifier for the institute from which they were found (UVic).

With both microsatellite and SNPs, any occasion where multiple markers were utilized from the same locus, the markers were given the locus name for mapping purposes. Locus names were assigned with a short identifier of the marker type (SNP or MI) followed by the word locus and a unique contig number (see Additional file 2: Table S1 and Additional file 3: Table S2 for contig numbers). Furthermore, should two markers from the same contig be useful in the same mapping parent, each marker was used to create a representative locus marker, with one marker filling in any missing data from the other. Each contig sequence used to design the SNP or microsatellite primers was input into an NCBI BLASTx search. Should the contig have a significant BLAST result, an abbreviation for the protein product was enclosed in square brackets, and joined to the original name (either marker or locus as necessary) by an underscore.

### Linkage analysis

Analysis of linkage in this study was performed using programs contained within the LINKMFEX package, v 2.3 (R. Danzmann, University of Guelph, http://www.uoguelph.ca/~rdanzman/software.htm) following the standard protocol (LOD threshold = 3.0). Markers with more than 15% of genotypes missing were omitted from the analysis. Merged sex-specific maps were produced followed by an estimated merged map using the MERGE programs in the LINKMFEX package. Should an insufficient number of common markers be mapped to linkage groups to be merged, the more complete linkage group was taken as a representative for the sex. Maps were visualized using the program MAPCHART [68].

#### *Anoplopoma fimbria* vs. *Gasterosteus aculeatus* (threespine stickleback)

Each of the contig sequences (minus extended repeats) used to design the primers for the markers linked to the map were input into the UCSC Genome Bioinformatics DNA Blat server (http://genome.ucsc.edu/cgi-bin/hgBlat?command=start; [69,70]) using the stickleback Feb. 2006 (Broad/gasAcu1) assembly. The hit with the highest score (min = 100, default BLAT score) was determined to be the most likely ortholog in the threespine stickleback, and chromosome number and position along the chromosome were retained. If contig sequences showed no BLAT hits, contigs were BLASTed to TSA database, and the top TSA hit BLATed against the *G. aculeatus* genome (min score = 150).

### GBS library

The GBS library was prepared following the protocol of Elshire et al. [21] utilizing the *Ape*KI restriction enzyme and the 96 barcodes suggested. 2 lanes of sample were run on a HiSeq 2000 with 100 bp paired-end reads (Illumina) and 48 individuals per lane. Resulting sequences were trimmed and markers were scored using the Stacks package [33]; see Additional file 7: Figure S3 for details. JELLYFISH v1.1 [48] was used to identify and count all 31mers in the dataset. The 31mers were searched for sequences found exclusively in males (family males >70% occurrence; unrelated males >60%; females <2) and females; the resulting kmers were mapped back to full-length reads and used to identify the potential male or female-specific polymorphisms.

### Genome walking

Genome walking was used to expand the available sequence around the male-specific markers, as well as the gene predicted to be between the male-specific markers using a protocol based on Siebert et al. [71] with primers from Rebrikov et al. [72]. Following three separate digestions of male and female sablefish DNA with restriction enzymes EcoRI, NdeI and BbsI (NEB), the genome walking adapter was ligated overnight at 16°C using T4 DNA ligase (NEB) in a 10 μl reaction. Following inactivation and dilution (1/10), the samples underwent PCR with primer P1 (5’-CTAATACGACTCACTATAGGGC-3’) and a specific primer designed to the 100bp read of interest (Additional file 9); PCR mix of 1X GoTaq Flexi Colorless PCR buffer (Promega), 2.5 mM MgCl_2_ (Promega), 320 μM each dNTP (Promega), 0.5 μM each forward and reverse primers (IDT), 0.75U GoTaq Hot Start polymerase (Promega), and 5ng of DNA template made up to 30 μl with DNAse/RNAse free H_2_O (Gibco), and cycled 95°C for 3 min (without template), 72°C for 10minutes (including template), 21 cycles of 95°C for 30 s, 62°C −0.5°C per cycle for 30 s and 72°C for 4 min, 19 cycles of 95°C for 30 s, 52°C for 30 s, 72°C for 4 min, with a final extension of 72°C for 10 mins. PCR were diluted 1/10000 and used as template for the second round using nested primer NP1 (5’-TCGAGCGGCCGCCCGGGCAGGT-3’) and a nested specific primer. PCR was cycled as above without the initial 72°C for 10 minutes. Following Exo/FastAP treatment (Fermentas), samples were sequenced following the SNP sequencing protocol [56]. Sequencing reactions were purified by ethanol precipitation while sequencing was performed on ABI 3730, and data analyzed using Geneious V5.1.7. Resulting sequences were BLATed against the *Gasterosteus aculeatus* genome.

### Identification of sex-specific markers and sequences

All primer sequences used in identification of Sex-specific markers can be found in Additional file 9. PCR primers were designed to sequences containing sex-linked polymorphisms, as well as genes predicted to be adjacent and between these markers based on the stickleback BLAT. PCRs were performed to amplify the sequence between the markers and genes, looking for sex-specific amplicons; long PCRs were performed following the Phusion (NEB) protocol with 35 cycles and 15 s/kb. Once sex-specific size differences were discovered, primers were designed to fully amplify across the region, with amplification and sequencing performed following Genome Walking PCR protocol, with 2 min extensions. Multiple bands were purified through gel extraction using the QIAquick Gel extraction kit (Qiagen). All 53 unrelated, mature sablefish were sequenced for the observed DNA inserts to confirm sex-specificity. Time-series samples were sexed using the X-insertion primers using the genomic DNA as template, while amplification on cDNA samples utilized exon-specific primers; see Additional file 9 for sequences and reaction conditions.

## Competing interests

The authors declare that they have no competing interests.

## Authors’ contributions

EBR performed EST library preparation and sequencing, primer evaluations, SNP sequencing and analysis, microsatellite genotyping and analysis, linkage mapping, mitochondrial genome analysis, comparative synteny analyses, GBS library construction, genome walking, design and testing of sex-specific primers and drafting of the manuscript. AMM performed EST library preparation and sequencing, microsatellite genotyping and analysis. DSS performed microsatellite primer design and genotyping. SGJ designed SNP primers and BLAT analysis, KRVS prepared samples for EST library and RNAseq and performed larval analysis, DRM identified sex-specific sequences in GBS dataset, JSL assembled and analyzed expressed sequences, WAP performed mitogenome sequencing. RSAM, GMM, AED performed microsatellite genotyping. BC produced and collected sablefish samples for analysis. BFK contributed to experimental design and analysis. All authors read and approved the final manuscript.

## Supplementary Material

Additional file 1: Figure S1 Sablefish mitochondrial genome. A graphical representation of the Sablefish mitochondrial genome, including the relative placement of the 13 genes, 22 tRNA and 2 rRNAs. Click here for file

Additional file 2: Table S1 Sablefish Microsatellite Primers. Details for all primers designed that successfully targeted polymorphic microsatellites. Primer sequences are detailed, as well as design sequence, BLAST and BLAT (stickleback) ID and repeat motif are given. Click here for file

Additional file 3: Table S2 Sablefish SNP Primers. Details for all primers designed that successfully targeted one or more polymorphic SNPs. Primer sequences are detailed, as well as design sequence, BLAST and BLAT (stickleback) ID, and total number of SNPs identified between the two families. Click here for file

Additional file 4: Table S3 Sablefish SNPs. List of all identified SNPs and their position within the surrounding sequence. Click here for file

Additional file 5: Figure S2 Sablefish linkage Map – Male, female and merged maps. All 24 linkage groups are presented. Each triplicate displays the merged linkage group in the middle, with the male-specific and female-specific linkage map to the left and right respectively. Click here for file

Additional file 6: Table S4 SNP markers identified through Genotyping-by-sequencing. List of SNPs identified through Genotyping-by-sequencing and parameters used in Stacks. Click here for file

Additional file 7: Figure S3 *Gsdf* and upstream promoter region. Sequences for Genbank:KC623942 and Genbank:KC623943 masked to show major features including sex-specific sequences, *gsdf*, sex-specific sequences and repeat elements. Click here for file

Additional file 8: Table S5 Mitochondrial genomes used in the phylogenetic analysis of the Scorpaeniformes and closely related Gasterosteiformes and Perciformes. All mitochondrial genome accession numbers used in the phylogenetic analysis. Accession numbers not included in Kawahara et al. [35] are marked in bold. Click here for file

Additional file 9 **Primers and relevant information for Sex-specific amplifications.** All primer sequences used to amplify and sequence the region containing the sex-specific sequences are given, as well as relevant information such as annealing temperatures and multiplexing strategies. Click here for file

## References

[B1] Smith WL Wheeler WC Polyphyly of the mail-cheeked fishes (Teleostei: Scorpaeniformes): evidence from mitochondrial and nuclear sequence data Mol Phylogenet Evol 2004 32 2 627 646 10.1016/j.ympev.2004.02.006 15223043

[B2] Shinohara G Imamura H Revisiting recent phylogenetic studies of “Scorpaeniformes” Ichthyol Res 2007 54 1 92 99 10.1007/s10228-006-0379-6

[B3] Kimura DK Shimada AM Shaw FR Stock structure and movement of tagged sablefish, *Anoplopoma fimbria,* in offshore northeast Pacific waters and the effects of El Nino-Southern Oscillation on migration and growth Fish Bull 1998 96 462 481

[B4] Allen MJ Smith GB Atlas and zoogeography of common fishes in the Bering Sea and Northeastern Pacific NOAA Technical Report NMFS 1988 66 1 151

[B5] Commercial Fisheries: Annual Landings[http://www.st.nmfs.noaa.gov/st1/commercial/landings/annual_landings.html]

[B6] Preliminary Summary Commercial Statistics 2011: Cumulative totals 2011 [http://www.pac.dfo-mpo.gc.ca/stats/comm/summ-somm/index-eng.htm]

[B7] Making a splash: Industry forecasts new aquaculture species will generate $880 million by 2020 [http://www.aquaculture.ca/files/archives.php]

[B8] Aggarwal RK Allainguillaume J Bajay MM Barthwal S Bertolino P Chauhan P Consuegra S Croxford A Dalton DL den Belder E Permanent genetic resources added to molecular ecology resources database 1 August 2010–30 September 2010 Mol Ecol Resour 2011 11 1 219 222 21429127 10.1111/j.1755-0998.2010.02944.x

[B9] McCraney WT Saski C Guyon J Isolation and characterization of 12 microsatellites for the commercially important sablefish, *Anoplopoma fimbria* Conservation Genet Resour 2012 4 2 415 417 10.1007/s12686-011-9563-8

[B10] Tripp-Valdez MA Garcia-de-Leon FJ Espinosa-Perez H Ruiz-Campos G Population structure of sablefish *Anoplopoma fimbria* using genetic variability and geometric morphometric analysis Population structure of sablefish J Appl Ichthyol 2012 28 4 516 523 10.1111/j.1439-0426.2012.01942.x

[B11] Nelson JS Fishes of the World 2006 Hoboken, NJ: John Wiley & Sons Inc

[B12] Liu ZJ Cordes JF DNA marker technologies and their applications in aquacultrue genetics Aquaculture 2004 238 1 37 10.1016/j.aquaculture.2004.05.027

[B13] Moen T Hayes B Baranski M Berg PR Kjoglum S Koop BF Davidson WS Omholt SW Lien S A linkage map of the Atlantic salmon (*Salmo salar*) based on EST-derived SNP markers BMC Genomics 2008 9 223 10.1186/1471-2164-9-223 18482444 PMC2405805

[B14] Lien S Gidskehaug L Moen T Hayes BJ Berg PR Davidson WS Omholt SW Kent MP A dense SNP-based linkage map for Atlantic salmon (*Salmo salar*) reveals extended chromosome homeologies and striking differences in sex-specific recombination patterns BMC Genomics 2011 12 615 10.1186/1471-2164-12-615 22182215 PMC3261913

[B15] Kucuktas H Wang S Li P He C Xu P Sha Z Liu H Jiang Y Baoprasertkul P Somridhivej B Construction of genetic linkage maps and comparative genome analysis of catfish using gene-associated markers Genetics 2009 181 4 1649 1660 10.1534/genetics.108.098855 19171943 PMC2666527

[B16] Zheng X Kuang Y Zhang X Lu C Cao D Li C Sun X A genetic linkage map and comparative genome analysis of common carp (*Cyprinus carpio* L) using microsatellites and SNPs Mol Genet Genomics 2011 286 3–4 261 277 21870156 10.1007/s00438-011-0644-x

[B17] Xia JH Liu F Zhu ZY Fu J Feng J Li J Yue GH A consensus linkage map of the grass carp (*Ctenopharyngodon idella*) based on microsatellites and SNPs BMC Genomics 2010 11 135 10.1186/1471-2164-11-135 20181260 PMC2838847

[B18] Reid DP Smith C-A Rommens M Blanchard B Martin-Robichaud D Reith M A Genetic Linkage Map of Atlantic Halibut (*Hippoglossus hippoglossus* L) Genetics 2007 177 1193 1205 10.1534/genetics.107.075374 17720928 PMC2034623

[B19] Franch R Louro B Tsalavouta M Chatziplis D Tsigenopoulos CS Sarropoulou E Antonello J Magoulas A Mylonas CC Babbucci M A genetic linkage map of the hermaphrodite teleost fish *Sparus aurata* L Genet Mol Biol 2006 174 853 861 10.1534/genetics.106.059014 PMC1602104 16951080

[B20] Amores A Catchen J Ferrara A Fontenot Q Postlethwait JH Genome evolution and meiotic maps by massively parallel DNA sequencing: spotted gar, an outgroup for the teleost genome duplication Genetics 2011 188 799 808 10.1534/genetics.111.127324 21828280 PMC3176089

[B21] Elshire RJ Glaubitz JC Sun Q Poland JA Kawamoto K Buckler ES Mitchell SE A Robust, simple genotyping-by-sequencing (GBS) approach for high diversity species PLoS One 2011 6 5 e19379 10.1371/journal.pone.0019379 21573248 PMC3087801

[B22] Matsuda M Nagahama Y Shinomiya A Sato T Matsuda C Kobayashi T Morrey CE Shibata N Asakawa S Shimizu N DMY is a Y-specific DM-domain gene required for male development in the medaka fish Nature 2002 417 6888 559 563 10.1038/nature751 12037570

[B23] Matsuda M Sato T Toyazaki Y Nagahama Y Hamaguchi S Sakaizumi M *Oryzias curvinotus* has DMY, a gene that is required for male development in the medaka, *O. latipes* Zoolog Sci 2003 20 2 159 161 10.2108/zsj.20.159 12655179

[B24] Hattori RS Murai Y Oura M Masuda S Majhi SK Sakamoto T Fernandino JI Somoza GM Yokota M Strüssmann CA A Y-linked anti-Müllerian hormone duplication takes over a critical role in sex determination Proc Natl Acad Sci 2012 109 8 2955 2959 10.1073/pnas.1018392109 22323585 PMC3286941

[B25] Yano A Guyomard R Nicol B Jouanno E Quillet E Klopp C Cabau C Bouchez O Fostier A Guiguen Y An immune-related gene evolved into the master sex-determining gene in rainbow trout, *Oncorhynchus mykiss* Current biology: CB 2012 22 15 1423 1428 10.1016/j.cub.2012.05.045 22727696

[B26] Yano A Nicol B Jouanno E Quillet E Fostier A Guyomard R Guiguen Y The sexually dimorphic on the Y-chromosome gene (sdY) is a conserved male-specific Y-chromosome sequence in many salmonids Evolutionary Applications 2012 2012 n/a-n/a 10.1111/eva.12032 PMC3673476 23745140

[B27] Myosho T Otake H Masuyama H Matsuda M Kuroki Y Fujiyama A Naruse K Hamaguchi S Sakaizumi M Tracing the emergence of a novel sex-determining gene in medaka, *Oryzias luzonensis* Genetics 2012 191 1 163 170 10.1534/genetics.111.137497 22367037 PMC3338257

[B28] Kamiya T Kai W Tasumi S Oka A Matsunaga T Mizuno N Fujita M Suetake H Suzuki S Hosoya S A trans-species missense SNP in *Amhr2* is associated with sex determination in the tiger pufferfish, *Takifugu rubripes* (Fugu) PLoS Genet 2012 8 7 e1002798 10.1371/journal.pgen.1002798 22807687 PMC3395601

[B29] Lee B-Y Kocher TD Liu Z Comparative genomics and positional cloning Aquaculture Genome Technologies 2007 Oxford, UK: Blackwell Publishing Ltd 323 335

[B30] Liu S Rexroad CE 3rd Couch CR Cordes JF Reece KS Sullivan CV A microsatellite linkage map of striped bass (*Morone saxatilis*) reveals conserved synteny with the three-spined stickleback (*Gasterosteus aculeatus*) Marine Biotechnol 2012 14 2 237 244 10.1007/s10126-011-9407-2 21968826

[B31] Palti Y Genet C Gao G Hu Y You FM Boussaha M Rexroad CE 3rd Luo MC A second generation integrated map of the rainbow trout (*Oncorhynchus mykiss*) genome: analysis of conserved synteny with model fish genomes Marine Biotechnol 2012 14 3 343 357 10.1007/s10126-011-9418-z 22101344

[B32] Sarropoulou E Nousdili D Magoulas A Kotoulas G Linking the genomes of nonmodel teleosts through comparative genomics Marine Biotechnol 2008 10 3 227 233 10.1007/s10126-007-9066-5 18297360

[B33] Catchen JM Amores A Hohenlohe P Cresko W Postlethwait JH *Stacks*: building and genotyping Loci *De Novo* from short-read sequences G3: Genes, Genomes, Genetics 2011 1 3 171 182 10.1534/g3.111.000240 PMC3276136 22384329

[B34] Pereira SL Mitochondrial genome organization and vertebrate phylogenetics Genet Mol Biol 2000 23 4 745 752 10.1590/S1415-47572000000400008

[B35] Kawahara R Miya M Mabuchi K Lavoue S Inoue JG Satoh TP Kawaguchi A Nishida M Interrelationships of the 11 gasterosteiform families (sticklebacks, pipefishes, and their relatives): a new perspective based on whole mitogenome sequences from 75 higher teleosts Mol Phylogenet Evol 2008 46 1 224 236 10.1016/j.ympev.2007.07.009 17709262

[B36] Kawahara R Miya M Mabuchi K Near TJ Nishida M Stickleback phylogenies resolved: evidence from mitochondrial genomes and 11 nuclear genes Mol Phylogenet Evol 2009 50 2 401 404 10.1016/j.ympev.2008.10.014 19027080

[B37] Imamura H Yabe M Demise of the Scorpaeniformes (Actinopterygii: Percomorpha): an alternative phylogenetic hypothesis Bulletin of Fisheries Sciences Hokkaido University 2002 53 3 107 128

[B38] Setiamarga DHE Miya M Yamanoue Y Azuma Y Inoue JG Ishiguro NB Mabuchi K Nishida M Divergence time of the two regional medaka populations in Japan as a new time scale for comparative genomics of vertebrates Biol Lett 2009 5 6 812 816 10.1098/rsbl.2009.0419 19586967 PMC2827986

[B39] Near TJ Eytan RI Dornburg A Kuhn KL Moore JA Davis MP Wainwright PC Friedman M Smith WL Resolution of ray-finned fish phylogeny and timing of diversification Proc Natl Acad Sci 2012 109 34 13698 13703 10.1073/pnas.1206625109 22869754 PMC3427055

[B40] Rexroad CE 3rd Palti Y Gahr SA Vallejo RL A second generation genetic map for rainbow trout (*Oncorhynchus mykiss*) BMC Genet 2008 9 74 19019240 10.1186/1471-2156-9-74 PMC2605456

[B41] Singer A Perlman H Yan Y Walker C Corley-Smith G Brandhorst B Postlethwait J Sex-specific recombination rates in Zebrafish (*Danio rerio*) Genetics 2002 160 2 649 657 11861568 10.1093/genetics/160.2.649 PMC1461993

[B42] Phillips RB Faber-Hammond J Luckenbach JA The sablefish (*Anoplopoma fimbria*) karyotype including the location of 5S and 18S rDNA and information on cell culture conditions Aquacult Res 2012 A Epub ahead of print 10.1111/j.1365-2109.2012.03177.x

[B43] Jaillon O Aury JM Brunet F Petit JL Stange-Thomann N Mauceli E Bouneau L Fischer C Ozouf-Costaz C Bernot A Genome duplication in the teleost fish *Tetraodon nigroviridis* reveals the early vertebrate proto-karyotype Nature 2004 431 7011 946 957 10.1038/nature03025 15496914

[B44] Davey JW Hohenlohe PA Etter PD Boone JQ Catchen JM Blaxter ML Genome-wide genetic marker discovery and genotyping using next-generation sequencing Nat Rev Genet 2011 12 7 499 510 10.1038/nrg3012 21681211

[B45] Peichel CL Ross JA Matson CK Dickson M Grimwood J Schmutz J Myers RM Mori S Schluter D Kingsley DM The master sex-determination locus in threespine sticklebacks is on a nascent Y chromosome Current biology: CB 2004 14 16 1416 1424 10.1016/j.cub.2004.08.030 15324658

[B46] Ross JA Urton JR Boland J Shapiro MD Peichel CL Turnover of sex chromosomes in the stickleback fishes (Gasterosteidae) PLoS Genet 2009 5 2 e1000391 10.1371/journal.pgen.1000391 19229325 PMC2638011

[B47] Smith EK Guzmán JM Luckenbach JA Molecular cloning, characterization, and sexually dimorphic expression of five major sex differentiation-related genes in a Scorpaeniform fish, sablefish (*Anoplopoma fimbria*) Comp Biochem Physiol B Biochem Mol Biol 2013 165 2 125 137 10.1016/j.cbpb.2013.03.011 23507626

[B48] Marcais G Kingsford C A fast, lock-free approach for efficient parallel counting of occurrences of k-mers Bioinformatics 2011 27 6 764 770 10.1093/bioinformatics/btr011 21217122 PMC3051319

[B49] de Boer JG Yazawa R Davidson WS Koop BF Bursts and horizontal evolution of DNA transposons in the speciation of pseudotetraploid salmonids BMC Genomics 2007 8 422 10.1186/1471-2164-8-422 18021408 PMC2198921

[B50] Kuraku S Qiu H Meyer A Horizontal transfers of Tc1 elements between teleost fishes and their vertebrate parasites, lampreys Genome Biol Evol 2012 4 9 817 824 10.1093/gbe/evs069 PMC3516227 22887124

[B51] Sawatari E Shikina S Takeuchi T Yoshizaki G A novel transforming growth factor-beta superfamily member expressed in gonadal somatic cells enhances primordial germ cell and spermatogonial proliferation in rainbow trout (*Oncorhynchus mykiss*) Dev Biol 2007 301 1 266 275 10.1016/j.ydbio.2006.10.001 17109839

[B52] Gautier A Le Gac F Lareyre J-J The gsdf gene locus harbors evolutionary conserved and clustered genes preferentially expressed in fish previtellogenic oocytes Gene 2011 472 1–2 7 17 21047546 10.1016/j.gene.2010.10.014

[B53] Yoshimoto S Ikeda N Izutsu Y Shiba T Takamatsu N Ito M Opposite roles of DMRT1 and its W-linked paralogue, DM-W, in sexual dimorphism of *Xenopus laevis*: implications of a ZZ/ZW-type sex-determining system Development 2010 137 15 2519 2526 10.1242/dev.048751 20573695

[B54] Smith CA Roeszler KN Ohnesorg T Cummins DM Farlie PG Doran TJ Sinclair AH The avian Z-linked gene DMRT1 is required for male sex determination in the chicken Nature 2009 461 7261 267 271 10.1038/nature08298 19710650

[B55] Alderdice DF Jensen JOT Velsen FPJ Preliminary trials on incubation of sablefish eggs (*Anoplopoma fimbria*) Aquaculture 1988 69 3–4 271 290

[B56] Messmer AM Rondeau EB Jantzen SG Lubieniecki KP Davidson WS Koop BF Assessment of population structure in Pacific *Lepeophtheirus salmonis* (Kroyer) using single nucleotide polymorphism and microsatellite genetic markers Aquaculture 2011 320 3–4 183 192

[B57] Sambrook J Russell DW Molecular Cloning: A Laboratory Manual 2001 3 Cold Spring Harbor, NY: Cold Spring Harbor Laboratory Press

[B58] Koop BF von Schalburg KR Leong J Walker N Lieph R Cooper GA Robb A Beetz-Sargent M Holt RA Moore R A salmonid EST genomic study: genes, duplications, phylogeny and microarrays BMC Genomics 2008 9 545 10.1186/1471-2164-9-545 19014685 PMC2628678

[B59] Rise ML von Schalburg KR Brown GD Mawer MA Devlin RH Kuipers N Busby M Beetz-Sargent M Alberto R Gibbs AR Development and application of a salmonid EST database and cDNA microarray: data mining and interspecific hybridization characteristics Genome Res 2004 14 3 478 490 10.1101/gr.1687304 14962987 PMC353236

[B60] Ewing B Green P Base-calling of automated sequencer traces using phred, II. Error probabilities Genome Res 1998 8 3 186 194 9521922

[B61] Ewing B Hillier L Wendl MC Green P Base-calling of automated sequencer traces using phred, I. Accuracy assessment Genome Res 1998 8 3 175 185 10.1101/gr.8.3.175 9521921

[B62] Li R Zhu H Ruan J Qian W Fang X Shi Z Li Y Li S Shan G Kristiansen K De novo assembly of human genomes with massively parallel short read sequencing Genome Res 2010 20 2 265 272 10.1101/gr.097261.109 20019144 PMC2813482

[B63] Miya M Nishida M Organization of the mitochondrial genome of a deep-sea fish, *Gonostoma gracile* (Teleostei: Stomiiformes): first example of transfer RNA gene rearrangements in bony fishes Marine Biotechnol 1999 1 5 416 426 10.1007/PL00011798 10525676

[B64] Wyman SK Jansen RK Boore JL Automatic annotation of organellar genomes with DOGMA Bioinformatics 2004 20 17 3252 3255 10.1093/bioinformatics/bth352 15180927

[B65] Rozen S Skaletsky H Primer3 on the WWW for general users and for biologist programmers Methods in molecular biology (Clifton, NJ) 2000 132 365 386 10.1385/1-59259-192-2:365 10547847

[B66] Benson G Tandem repeats finder: a program to analyze DNA sequences Nucleic Acids Res 1999 27 2 573 580 10.1093/nar/27.2.573 9862982 PMC148217

[B67] Jackson TR Ferguson MM Danzmann RG Fishback AG Ihssen PE O'Connell M Crease TJ Identification of two QTL influencing upper temperature tolerance in three rainbow trout (*Oncorhynchus mykiss*) half-sib families Heredity 1998 80 143 151 10.1046/j.1365-2540.1998.00289.x

[B68] Voorrips RE MapChart: software for the graphical presentation of linkage maps and QTLs J Hered 2002 93 1 77 78 10.1093/jhered/93.1.77 12011185

[B69] Fujita PA Rhead B Zweig AS Hinrichs AS Karolchik D Cline MS Goldman M Barber GP Clawson H Coelho A The UCSC Genome Browser database: update 2011 Nucleic Acids Res 2011 39 supplement 1 D876 D882 20959295 10.1093/nar/gkq963 PMC3242726

[B70] Kent WJ Sugnet CW Furey TS Roskin KM Pringle TH Zahler AM David H The human genome browser at UCSC Genome Res 2002 12 6 996 1006 12045153 10.1101/gr.229102 PMC186604

[B71] Siebert PD Chenchik A Kellogg DE Lukyanov KA Lukyanov SA An improved PCR method for walking in uncloned genomic DNA Nucleic Acids Res 1995 23 6 1087 1088 10.1093/nar/23.6.1087 7731798 PMC306810

[B72] Rebrikov DV Desai SM Siebert PD Lukyanov SA Shimkets RA Suppression subtractive hybridization Methods in Molecular Biology Vol. 258, Gene expression profiling: methods and protocols 2004 Totowa, NJ: Humana press 107 134 10.1385/1-59259-751-3:107 14970460

