# Supplementary figures and images for: Genomics of sablefish (Anoplopoma fimbria): expressed genes, mitochondrial phylogeny, linkage map and identification of a putative sex gene
Source: BMC Genomics. 2013 Jul 6;14:452. doi: 10.1186/1471-2164-14-452 (PMC3708741; doi:10.1186/1471-2164-14-452)

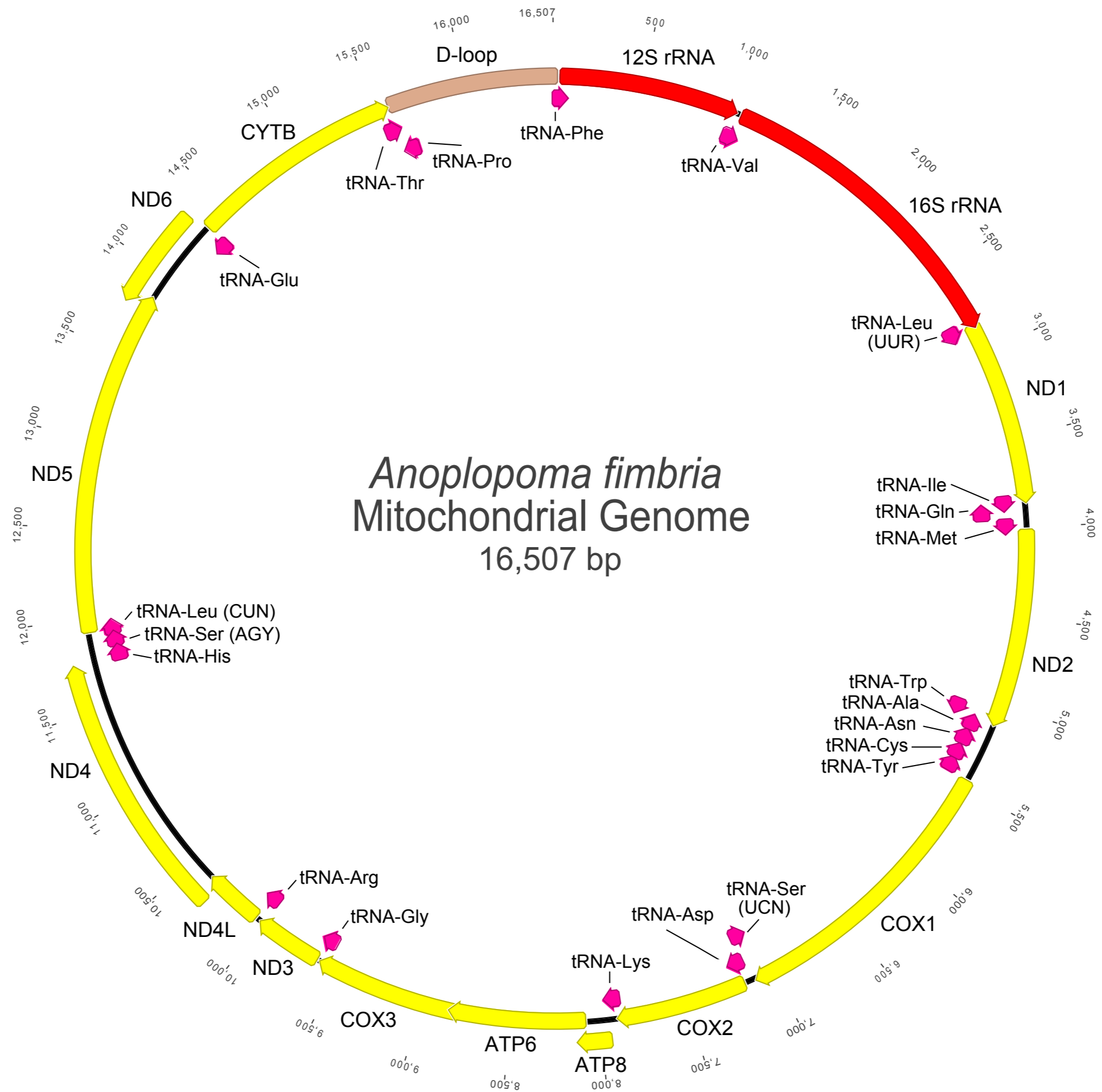

Supplement: Additional file 1: Figure S1 — Sablefish mitochondrial genome. A graphical representation of the Sablefish mitochondrial genome, including the relative placement of the 13 genes, 22 tRNA and 2 rRNAs. [file 1471-2164-14-452-S1.pdf]

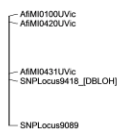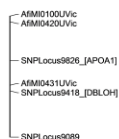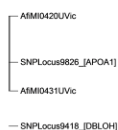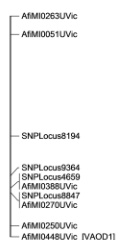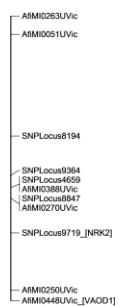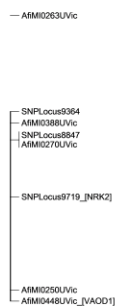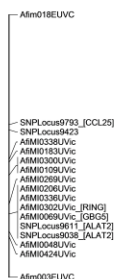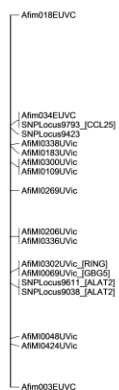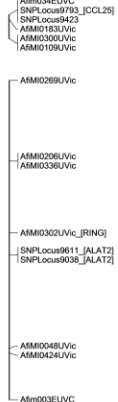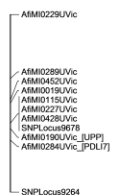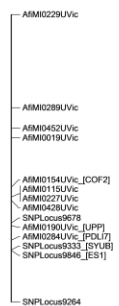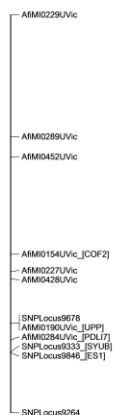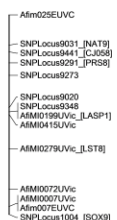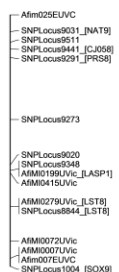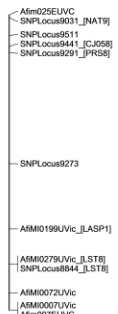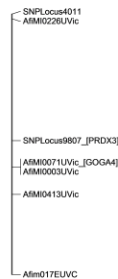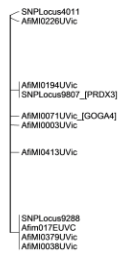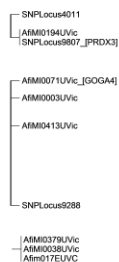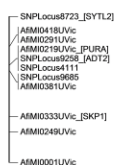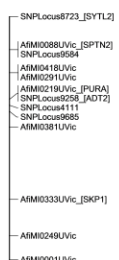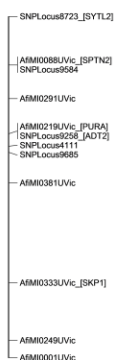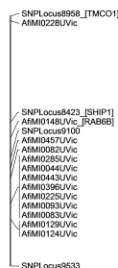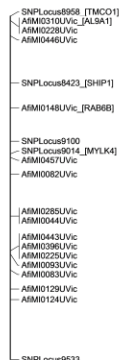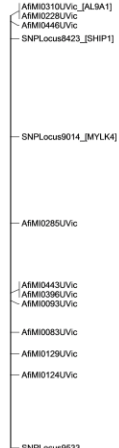

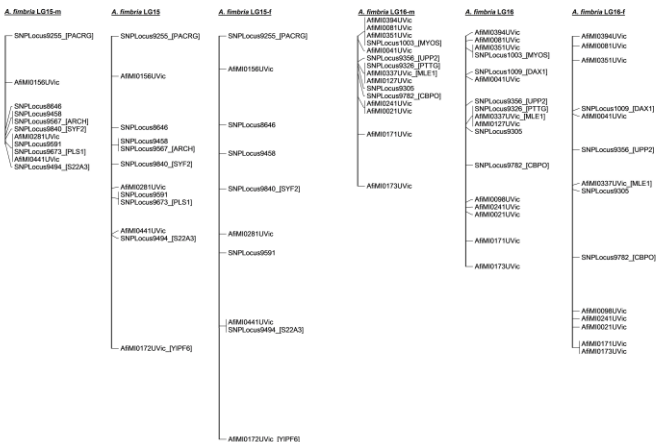

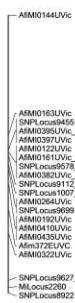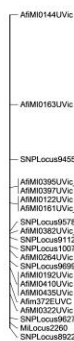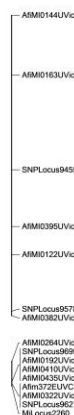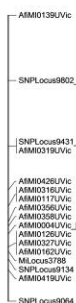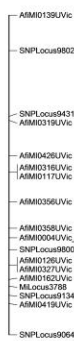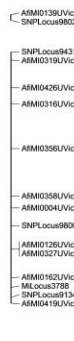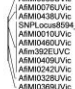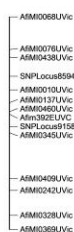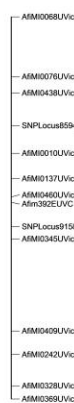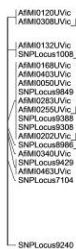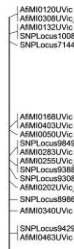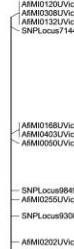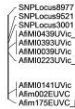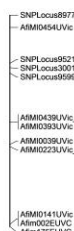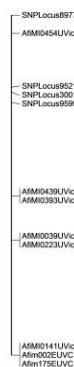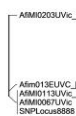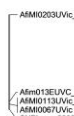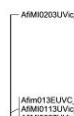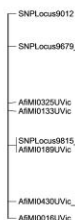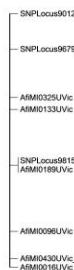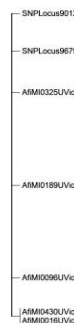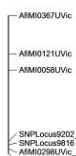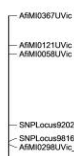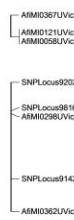

Supplement: Additional file 5: Figure S2 — Sablefish linkage Map – Male, female and merged maps. All 24 linkage groups are presented. Each triplicate displays the merged linkage group in the middle, with the male-specific and female-specific linkage map to the left and right respectively. [file 1471-2164-14-452-S5.pdf]
