# Supplementary material for: Genomics of sablefish (Anoplopoma fimbria): expressed genes, mitochondrial phylogeny, linkage map and identification of a putative sex gene
Source: BMC Genomics. 2013 Jul 6;14:452. doi: 10.1186/1471-2164-14-452 (PMC3708741; doi:10.1186/1471-2164-14-452)
Supplement: Additional file 7: Figure S3 — Gsdf and upstream promoter region. Sequences for Genbank:KC623942 and Genbank:KC623943 masked to show major features including sex-specific sequences, gsdf, sex-specific sequences and repeat elements. [file 1471-2164-14-452-S7.doc]

Polymorphisms between X and Y

Large Repeat 1

Large Repeat 2

Inverted relative to repeat

Small repeat (same orientation)

Self inversion (hairpin capable)

Coding region

X-specific sequence

TAG10:

> KC623942 – X chromosome

GTTTTATAAAGGCTCTTTGTGATCCCAGCCAGGAGCAGCTGCCGGTGGAGTGACTCCCA

GCAGCACCACAACATGGAAGTGGTGGGAGACGTCTGATACTCAAGGACTCCGCTCCATT

CATGCATGGTTATAGGATGTCAAGAGCTGCAGTATCCCACAGTTTATGTGTGTGTGTGT

TTGTGTGTGTACATACATACTGTACTGTGGGTAACCACCTTTATATATACTCAAACACA

CAACTGTGTACATGGGTATTTTTCTCACATACACCCATAGATAATATACACACCATTTT

TATCTGTAGTCAATGTCCAACTTCTCTTAGAGTTTGACACTATTTTTGTAGTGTCAAAA

TGGACAATGGAGGAAAAGTATATATCAGCTAATGCTTATTATCAGAATTATTTTATTCT

TGCTTTTGTTCTTTGATGGGATTTGTTTACAGTTTGTGGCTAGTTGGGTATATGTTTGT

GCATGTCATACCAATCAGCACTGACATTCTGCTGTATTTTGTGGTTTGTCATTTATGAA

AGTTTTCCAGCACTCCGAAGTTGATTCCTGAAGATAATTTAAAAAGTGGGATCTTCACT

GTGTTGTTGAGGCCTGTAGAACATTTTTGGGTATGATGCCCAGATAGGTTGTTACCAAG

CACACAAAGTGCTTTAGGGTTCAGAAAGATTTGACAGTGAAGGAAGACGTATTCAGAAG

AGGTCATAACAACGTTATCGTTTTCAGATTTTTTCTCCCCATAAGAGTTATCCCTTCGA

ACAAATAATTCTACACGATCAGCTCTTCGGCTCAGGTATTGGCCGATTAATCGTCGTCC

AAACTATCGTTATGATATTATATTTAAGATCCACTAGCAGTCGACCCCTAGTATTCATA

TCATGCCGTGAACCAGTTTTGGCCTCAGCGAGGAGTCGATAACATGTTAACAAAAATAA

ATACTCTTCTATTCTTCCCATGCTTATCTGGATTTGCTGATAAAACGCAAAAGAAAAGT

CTTAGCTACTGTAGCAGGTGCTTTGGTGGCTCCTGCACTCTCTGAATGTGGATTACGGT

ATTAGCAACAGCGTGCCAGTGGATCACTCATTACAAACTCATGCTGTGCAGCCAAATAT

TGCGTAATTAAAGCTAGGGTGGGTATTAATTAATTAATTTCTTTACTGTAATATTTGGC

AAAACTGTCCTAATAGCCAGTCAGCTATATGTAGGTGAAGTGCTCTGAGAATATCTGCT

CATAACTTTGCTCTCCCCTGCCTCTGTGGGCCGCACCCAAAAATTGCCACCGCTCATGT

ACACTTTGACCAATCAGAGCGAGGCTCCGATTCTCCGTTCTTATTGGCTGTGGGACTGT

CCGGTCACCTTGGCAACGACGTTGGTGCGTCCCTGCGTGTTTCGTCTTCCTATCAATGT

AACTGAATTCACATGAACGCGCATAACTGACGTTCGGTGGGAGGAGCTACAGCAGGTAGCGTGGCAGGGAGAGGCCAGAGAGCACGCGACGGAATCTCAACGGCTGTTTTCGCCCACC

CTAGCTTTAAGTGTCCAAAAATAACCAATAGAATCAGAATGAGGTATTTTCATTTGATG

GTACAGTGCAGCACACAAGCTGTTTAGCTCCCAGAAGAGCGAGAACAAGCTGATGCAGA

ATATATACAGAGTTTCAAGAAGAAACACCTCAAAACATAATGTTGACAATGTTGACAGA

ACTGAACATTATGAACTACGCATAGATAGATGTTATTGCAGGGCTGTTGGATTTCATTT

GAGTGTATAGATATACTTAATAAAGTGGTCACTAAGCGTATATGATACTGGTATTGTGA

AATAATATAACTTGAATACCAGTTAAGCTACTTATGTGTCGTCAGAAGGGGAAATTGTA

TAATAGGAAGGAAGGAATTCGGTAACAATTTGTGACAAGTGTGGTTGGCATGAATTGAG GCACACGTTGGGTTGTCTTTGGATTTCTTGTTAGGTGAGTCCTGATTTTCACCATGTCG

TAATGCTTCTCATCCCTGAAGATTTCTGCCTTTCATGCCCAACCAAAAAGTGGTAGTTT

AAACAGATACTGATAACTTCATGTGCAGAATTAAATTTGCAGCTCGTGTTTTATGGTTA

GGTGGCTTATTATTGAAAACATATTAACAAAAATAAATACTCTTCTATTCTTCCCATGC

TTATCTGGATTTGCTGATAAAACGCAAAAGAAAAGTCTTAGCTACTGTAGCAGGTGCTT

TGGTGGCTCCTGCACTCTCTGGATGTGGATTATTATTAGTATTATTAGCAGCAGCGTGC

CAGTGGATCACCACTCATTACTTGAATACCAATTAAGATACTTATGTGTCGTCAGAAGG

GGAAACTGTATAAAGGAATTCACTAACGATTTGTGACAAGTGTGGTTGGCTGGAATTGA

CGCACACGTTGGGTTGTCTTTGGATTTCTTGTTGTAACAAATCAACAAGTTTAAAACGG

AAAGCATATGCTAAAGTGGTATGCCTATGAACAAATGTTGCGCTCTCACCTTTCATTCC

TGTCTGTTTGTGTAGAGTCTATTAGTCTCTCTTTCTTTCTCTCCTGGATCATCCTCCCT

GCGATCTTTCACACACAAAATGGGACAAGACAACAGTGTTAGATATCCCAAGGTCAAGC

TGTGTTACAAGAAGATAAAAACCCAGGTTCC**CAGG**ACCTGACAAACACTACCTAGAAGA

GCATCTCTGTCTGGTCACTCCTATCTCCTCCTCCAACGAGAAGCACAGAACTAATCACA

GCCCGAGACCGCCGCAAGTCTGGCTCAGAAAGCAGTTGATCTATAGATTGACCGTCCAG

CTCGCTCACCATGTCCTTTACCTTCGTTGTCACGACGATGCTTCTGAGCTCTTCAGTGG

TGAATACATTTGTCTTGCAGTCATCCAAGGAAGAACCTACAGCCTCTGCTAACTCTCCT

GTTTCCAATCCCAGGTCAGTATCAAACTTTATTTACCATTTTTCTGCTTTTGTTCGCAG

TGACATGTCTTCGTATGGAGGGAACAAATCTAAAACTGTTGTCCTCAAAAGCGCCCAAA

GAAATATGCAACATGTCACAGAGGTTATTATATTTTAAAATAGCCTCAGCATCAGATCA

CATTTCACAAGATGTTTTTGTGCTTAGACCTTTTTTAATTTGGGTTACAGATATATTTT

AGGTTATGGGTAGGATTCAGTGTCACTGTGACCATTTGGTTTTTTAAGAATGTTGTGCA

GAGATGTAACACACTGTTTAAGTTTCAGCAGTGCTATCAATTACTCTTCAACACAGCAT

TTCTTTCACTTGGTTTAGCCAAATGCCTGGAATGCTCTTCAGCTGTTCATTTAGTGCAT

TTAGAGAGCATGTCTTGCATTTGAGGCTACTAAATAAATGCTTGATTGGAGTGTAAACA

AAATATTTGTGAAGTGTCCTGGATTTCTTTTATTTCTATGTTTTTCCGTATAATATATT

TTTTAAAGTATGCAGTATGTAGATAATCTGAGTGACATATGGTGTTCAGGTGCCAGGGG

GGGTCCCTGCAGTTCACACGGAAGAGCCTCCTCGGGGCTCTAAACTTGCAGACTGAGCC

ACAGCTGCGTGCTGGTGGGCTTGATGGTATCAGAGAGCACTGGAGGACCACCTTCAGCA

CCATCACTCACAGAGCCATGGACACAGCAGGTAAGTGGAAGCCAAAATTGAAAGAAACT

ACTGTAGAGCCCACATCTAGCCAGTTTAATCCATAGTGGCTGGGAGTCAAATCCAGCAA

AACGTGTCTTTTTTTCCATGTCATTCCAGTTCCAGCGGCCTCTGGCTACTCTGTGTCAC

CTAATGTTGGAAACAGTACGGGCCTGAGGTGCTGTTCTATGGCCACTGAGATCAGCATG

ACAGGTATGCAACAATGTGTGTGCATGCATATTGCCATGAAGAATTTCAGTTATGCATG

TACTGTAATATTCATTCTTGTGTTTGCAAGATCTGGGATGGGACAACTGGATTATCCAT

CCTGCCAGCCTAACCATTGTTCAGTGTGCACTCTGCAACCCCAAAGTGAACACTGGGCA

ATGCCCATCACCCCATGCCAATGTCCAGGATGCTGAATCACAGGTATAATTATTTAAGA

CTTTTAAATATTGTAATGATTGCCTCCAAACTCCTACATTTACATACATTGATACTTTG

ATTGTACTTTCCTCCATGTCCTACAACGTAAGTACAGTAGTTTAATCTATGTAATTAGT

TACTTTCCACCAATGTGCCTGAGTTTCAGTCTCTTGGAACTACGAACAGAATATAGTTT

TGAGCTTGTGCGTGTTTGGGAGGCGAAACCGCTGCCAATAGAAACGATTTCTGCTTCTG

CCAACACAAATGTACCAAATACAAGCTAAGACACTCCCCAAAGTTGGTCAATCAGAAAT

GAATGCAGTCACAGATATGTGTGCGAGTTTTAACCTAACATTTTGGCAGGCCACATTTG

TTTATTTTGTTAATCTCATTGGCATTTGCTAACATTATTTCTGTTATTTATCTACACTT

TTTCTTCGATAACTTGGTGTGAACATTAATTGACATCCACTAAAGCATGTATCAAATGC

ATTGCATTTTCTATTTTCCCATCAATATAAATGTATATGTTACATTAGAAAAAAAAAAA

AAAATCAATTCAAGTCATTCTTTTGTTCAAACTGTATTACCACGCCTTACTCCTCCTCT

CTTTGTTCCTCTTCAGGTGCCATGTTGTGAGCCCATCTCCCAGGAAATGGTTCCCGTCC

TCTACATGGATGAATTCAGCAACCTTGTAATTTCCTCCGTGCATCTGACCCGCAGCTGC

GGTTGTGATCGTGGCAACCTCCAGCCACCCAGCAGAGAGTAA

Polymorphisms between X and Y

Large Repeat 1

Large Repeat 2

Inverted relative to repeat partner

Small repeat (same orientation)

Self inversion (hairpin capable)

Coding region

Y-specific inserted sequence

Repeated start/end of Y-specific

TAG10:

> KC623943 – Y chromosome

GTTTTATAAAGGCTCTTTGTGATCCCAGCCAGGAGCAGCTGCCGGTGGAGTGACTCCCA

GCAGCACCACAACATGGAAGTGGTGGGAGACGTCTGATACTCAAGGACTCCGCTCCATT

CATGCATGGTTATAGGATGTCAGGAGCTGCAGTATCCCACAGTTTATGTGTGTGTGTGT

TTGTGTGTGTACATACATACTGTACTGTGGGTAACCACCTTTATATATACTCAAACACA

CAACTGTGTACATGGGTATTTTTCTCACATACACCCATAGATAATATACACACCATTTT

TATCTGTAGTCAATGTCCAACTTCTCTTAGAGTTTGACACTATTTTTGTAGTGTCAAAA

TGGACAATGGAGGAAAAGTATATATCAGCTAATGCTTATTATCAGAATTATTTTATTCT

TGCTTTTGTTCTTTGATGGGATTTGTTTACAGTTTGTGGCTAGTTGGGTATATGTTTGT

GCATGTCATACCAATCAGCACTGACATTCTGCTGTATTTTGTGGTTTGTCATTTATGAA

AGTTTTCCAGCACTCCGAAGTTGATTCCTGAAGATAATTTAAAAAGTGGGATCTTCACT

GTGTTGTTGAGGCCTGTAGAACATTTTTGGGTATGATGCCCAGATAGGTTGTTACCAAG

CACACAAAGTGCTTTAGGGTTCAGAAAGATTTGACAGTGAAGGAAGACGTATTCAGAAG

AGGTCATAACAACGTTATCGTTTTCAGATTATTTCTCCCCATAAGAGTTATCCCTTCGA

ACAAATAATTCTACACGATCAGCTCTTGGGCTCAGGTATTGGCCGATTAATCGTCGTCC

AAACTATCGTTATGATATTATATTTAAGATCCACTAGCAGTCGACCCCTAGTATTCATATCATGCCGTGAACCAGTTTTGGCCTCAGCGAGGAGTCGATAACATGTTAACAAAAATAA

ATACTCTTCTATTCTTCCCATGCTTATCTGGATTTGCTGATAAAACGCAAAAGAAAAGT

CTTAGCTACTGTAGCAGGTGCTTTGGTGGCTCCTGCACTCTCTGAATGTGGATTACGGT

ATTAGCAACAGCGTGCCAGTGGATCACTCATTACAAACTCATGCTGTGCAGCCAAATAT

TGCATAATTAA

GTGTCCAAAAATAACCAATAGAATCAGAATGAGGTATTTTCATTTGATG

GTACAGTGCAGCACACAAGCTGTTTAGCTCCCAGAAGAGCGAGAACAAGCTGATGCAGA

ATATATACAGAGTTTCAAGAAGAAACACCTCAAAACATAATGTTGACAATGTTGACAGA

ACTGAACATTATGAACTACGCATAGATAGATGTTATTGCAGGGCTGTTGGATTTCATTT

GAGTGTATAGATATACTTAATAAAGTGGTCACTAAGCGTATATGATACTGGTATTGTGA

AATAATATAACTTGAATACCAGTTAAGCTACTTATGTGTCGTCAGAAGGGGAAATTGTA

TAATAGGAAGGAAGGAATTCGGTAACAATTTGTGACAAGTGTGGTTGGCATGAATTGAG

GCACACATTGGGTTGTCTTTGGATTTCTTGTTAGGTGAGTCCTGATTTTCACCATGTCG

TAATGCTTCTCATCCCTGAAGATTTCTGCCTTTCATGCCCAACCAAAAAGTGGTAGTTT

AAACAGATACTGATAACTTCATGTGCAGAATTAAATTTGCAGCTCGTGTTTTATGGTTA

GGTGGCTTATTATTGAAAACATATTAACAAAAATAAATACTCTTCTATTCTTCCCATGC

TTATCTGGATTTGCTGATAAAACGCAAAAGAAAAGTCTTAGCTACTGTAGCAGGTGCTT

TGGTGGCTCCTGCACTCTCTGGATGTGGATTATTATTAGTATTATTAGCAGCAGCGTGC CAGTGGATCACCACTCATTACTTGAATACCAATTAAGATACTTATGTGTCGTCAGAAGG

CAGTGGTGTAGTCCAGGGTATACGGCGTATACCCACTTATTTTTCAGTCAGCATTGCGTATACCCACTTCTAAACCCCCCCAGATGCGCACCATTCAGTAGTATCTGCAAGCGAAATCGCGCACTTTTCCCCGGTGAAGCCATGACCCACGCTACTCTGCCACTTATTGGACTAATACTCGCTGCCTTTTACTGATTAGATTGGTTAACTGTAGGCATGAGTCATGACCGCATGACGACGAAATGAGCTCCAATCGGAGGGAGAGAAAGGCGGGTCATGCCGAAGTAAATATCGCGAAAGACTAATTTACTTTTACAAGTTTACTTTAAATGATTTTCTATAATAATCCGGTAAAATGAAGCGAGCTATCCAAACAAAAATTTCATTTGACAATTTCAAACGTCCTCGCGAATCAATATCAGCCAGCAGCTCAGCAGTTAATGTTAGCGATCGTACACCGGTACAGTTACCGTCGACGCCCGCTCTGCCCCCCGCCCCCGTCGGAGAGGAGGCTGTCAGTAAAGGAGATGCTGCCTCTCCGCCCACAGCCAAAGATGCCGGTACAACTTCGGATGAAGACGTCAACTACGGTAGGTTTCATAATAGTGGCACACTCTGGCCCATGCTATGTGTTGCGAATCAGCTGTTCAAAGACACCGTTTACAAAATAAATTACTGATTTGCCATGTGAGAGAATAAGTGTCATTCCAGGAATAAGGAATAGTTGTCTCTCACTAGTCAGTATTTGTTTAGTCATTTCATAATCCTTCCTCCCTGAGATCAAGCAGCAGAAATTATGTGCCAATGTAAGTAAACTAAAAAGAGTAGTCTCAAATATCACTGTTTCTAAAAAAGGGTGTTTAGGGCGTTAAAAAAAGGGCAAACATTTAGAGTATACCCACTTCTCCAGGGACCACTACACCACTGTCAGAAGG

GAAAACTGTATAAAGGAATTCACTAACGATTTGTGACAAGTGTGGTTGGCTGGAATTGA

CGCACACGTTGGGTTGTCTTTGGATTTCTTGTTGTAACAAATCAACAAGTTTAAAACGG

AAAGCATATGCTAAAGTGGTATGCCTATGAACAAATGTTGCGCTCTCACCTTTCATTCC

TGTCTGTTTGTGTAGAGTCTATTAGTCTCTCTTTCTTTCTCTCCTGGATCATCCTCCCT

GCGATCTTTCACACACAAAATGGGACAAGACAACAGTGTTAGATATCCCAAGGTCAAGC

TGTGTTACAAGAAGATAAAAACCCAGGTTCC**CAGG**ACCTGACAAACACTACCTAGAAGA

GCATCTCTGTCTGGTCACTCCTATCTCCTCCTCCAACGAGAAGCACAGAACTAATCACA

GCCCGAGACCGCCGCAAGTCTGGCTCAGAAAGCAGTTGATCTATAGATTGACCGTCCAG

CTCGCTCACCATGTCCTTTACCCTCGTTGTCACGACGATGCTTCTGAGCTCTTCAGTGG

TGAATACATTTGTCTTGCAGTCATCCAAGGAAGAACCTACAGCCTCTGCTAACTCTCCT

GTTTCCAATCCCAGGTCAGTATCAAACTTTATTTACCATTTTTCTGCTTTTGTTCGCAG

TGACATGTCTTCGTAAGGAGGGAACAAATCTAAAACTGTTGTCCTCAAAAGCGCCCAAA

GAAATATGCAACATGTCACAGAGGTTATTATATTTTAAAATAGCCTTAGCATCAGATCA

CATTTCACAAGATGTTCTTGTGCTGAGACCTTTTTTAATTTGGGTTACAGATATATTTT

AGGTTATGGGTAGGATTCAGTGTCACTGTGACCATTTGGTTTTTTAAGAATGTTGTGCA

GAGATGTAACACACTGTTTAAGTTTCAGCAGTGCTATCAATTACTCTTCAACACAGCAT

TTCTTTCACTTGGTTTAGCCAAATGCCTGGAATGCTCTTCAGCTGTTCATTTAGTGCAT

TTAGAGAGCATGTCTTGCATTTGAGGCTACTAAATAAATGCTTGATTGGAGTGTAAACA

AAATATTTGTGAAGTGTCCTGGATTTCTTTTATTTCTATGTTTTTCCGTATAATATATT

TTTTAAAGTATGCAGTATGTAGATAATCTGAGTGACATATGGTGTTCAGGTGCCAGGGG

GGGTCCCTGCAGTTCACACGGAAGAGCCTCCTCGGGGCTCTAAACTTGCAGACTGAGCC

ACAGCTGCGTGCTGGTGGGCTTGATGGTATCAGAGAGCACTGGAGGACCACCTTCAGCA

CCATCACTCACAGAGCCATGGACACAGCAGGTAAGTGGAAGCCAAAATTGAAAGAAACT

ACTGTAGAGCCCACATCTAGCCAGTTTAATCCATAGTGGCTGGGAGTCAAATCCAGCAA

AACGTGTCTTTTTTTCCATGTCATTCCAGTTCCAGCGGCCTCTGGCTACTCTGTGTCAC

CTAATGTTGGAAACAGTACGGGCCTGAGGTGCTGTTCTATGGCCACTGAGATCAGCATG

ACAGGTATGCAACAATGTGTGTGCATGCATATTGCCATGAAGAATTTCAGTTATGCATG

TACTGTAATATTCATTCTTGTGTTTGCAAGATCTGGGATGGGACAACTGGATTATCCAT

CCTGCCAGCCTAACCATTGTTCAGTGTGCACTCTGCAACCCCAAAGTGAACACTGGGCA

ATGCCCATCACCCCATGCCAATGTCCAGGATGCTGAATCACAGGTATAATTATTTAAGA

CTTTTAAATATTGTAATGATTGCCTCCAAACTCCTACATTTACATACATTGATACTTTG

ATTGTACTTTCCTCCATGTCCTACAACGTAAGTACAGTAGTTTAATCTATGTAATTAGT

TACTTTCCACCAATGTGCCTGAGTTTCAGTCTCTTGGAACTACGAACAGAATATAGTTT

TGAGCTTGTGCGTGTTTGGGAGGCGAAACCGCTGCCAATAGAAACGATTTCTGCTTCTG

CCAACACAAATGTACCAAATACAAGCTAAGACACTCCCCAAAGTTGGTCAATCAGAAAT

GAATGCAGTCACAGATATGTGTGCGAGTTTTAACCTAACATTTTGGCAGGCCACATTTG

TTTATTTTGTTAATCTCATTGGCATTTGCTAACATTATTTCTGTTATTTATCTACACTT

TTTCTTCGATAACTTGGTGTGAACATTAATTGACATCCACTAAAGCATGTATCAAATGC

ATTGCATTTTCTATTTTCCCATCAATATAAATGTATATGTTACATTAGAAAAAAAAAAA

AAAATCAATTCAAGTCATTCTTTTGTTCAAACTGTATTACCACGCCTTACTCCTCCTCT

CTTTGTTCCTCTTCAGGTGCCATGTTGTGAGCCCATCTCCCAGGAAATGGTTCCCGTCC

TCTACATGGATGAATTCAGCAACCTTGTAATTTCCTCTGTGCATCTGACCCGCAGCTGC

GGTTGTGATCGTGGCAACCTCCAGCCACCCAGCAGAGAGTAA
