# Supplementary material for: Genomics of sablefish (Anoplopoma fimbria): expressed genes, mitochondrial phylogeny, linkage map and identification of a putative sex gene
Source: BMC Genomics. 2013 Jul 6;14:452. doi: 10.1186/1471-2164-14-452 (PMC3708741; doi:10.1186/1471-2164-14-452)
Supplement: Additional file 9 — Primers and relevant information for Sex-specific amplifications. All primer sequences used to amplify and sequence the region containing the sex-specific sequences are given, as well as relevant information such as annealing temperatures and multiplexing strategies. [file 1471-2164-14-452-S9.doc]

**All primer sequences 5'->3'**

**All following PCR reactions contain the following unless otherwise stated:**

1X GoTaq Flexi Colorless PCR buffer (Promega), 2.5mM MgCl_2_ (Promega), 320µM each dNTP (Promega), 0.5 µM each forward and reverse primers (IDT), 0.75U GoTaq Hot Start polymerase (Promega), and 5ng of DNA template made up to 10µl with DNAse/RNAse free H_2_O (Gibco)

**Genome Walking: (Protocol described in text and references)**

**Tag 2: kmer assembly =** CAGCTGAAAAAAA[C]AACAACACACAAATCATTTAACACATTTCA

SF_MALE_TAG2_1R CAACACACAAATCATTTAACACA

SF_Male_TAG2_2R CAACCAAACATGTAGGATGAAC

SF_Male_TAG2_1F GTTCATCCTACATGTTTGGTTG

SF_Male_Tag2_2F TGTGTTAAATGATTTGTGTGTTG

Tag 2 is an inserted base found so far only in males, marked here by brackets []

GAGCGGCCGCCCGGGCAGGTATCAGGCCTCTGGATGGCAGGCTTGTCACAGAACAGTATTGTGTGTTTTT

GTGTGTGCTTCATGCAAAAAGAGAGGTGAAAAAGTGGAAACAGTTGAAAGATCTTCTAAGAACTTTGTGA

TTTTCAATATCTGAGACACTTAAAACAACCAAAATGCTGTGTGTCATGACCGATCACTGCCCTCAATCAG

TTCCTGAAATTTACTAAATTCAATCCCCAGATTTTCCAGCTTCTTTGACAGTACATTAAGTAAGTGCATG

TCATACATACTGCACTAAATACTATGTGTTCAATATCTAAATCCCGATATTGGAAATGTGTATTGAGAAA

GTCACAAAAGTAATGCTAATTTTAATTCTCTTTTTGTATCAACTAATTATAATGTTTTACATTATGCATA

TCATGATCTTAAGGTCCCAGTCTTTATCTACAATGTTAAAAGTCTAATGGTCAGCTCGTTCAGCAGTCCC

GAGCCTCCAGTTAGGTCTAAATGAAGTAAGATGATGTTGGTGTGCTCTGCGGGCAGACAGCCAGTAGCAT

AACATAACCACCCAGGGTGAGTGTCCAATAACAGACCTTTCTGCACAGCCTGTACTTCAGGCCTGCATTA

AATCATACACGTGCTGGTGTCCGTGCTCCAAGAGAAGCAGACACAGGCTGCTTGATTCTTTTGTGAAGCA

CTTTCCCGTGTGTTCATCCTACATGTTTGGTTGAAATGTGTTAAATGATTTGTGTGTTGTT [G] TTTTTTTC

AGCTGCAGGATAAGCTCTAAAACCTACATGTGACATTACAACCACTGCCAGCGCTGGGCTCTGTGTCATC

AAATCTGAACTCATGCACCAAAACTAGCACCGAGGTGGCATAGTTAGTCAGACTTTATGATGATCTACTT

CAGTATTTCTGCGCAGGAAAGTGAATCCTTGCCAGCTTCAGAGGTCCTTCTTTTTTAGTTGTTAACAAGG

TAAGTATCAATCATTCAAAATGGTGTGACTGTTTATTTATCTCTAAAAGAAATCATACATCATGTATATT

GTGTGTACTTGGTCTAATTTCTGTTTGCTTCAAAGTAAAAGTGTTTTGTGCTCTATCCACAATAACCTTT

GTCACATATATGTCCACCATTCCTGTTTAAATTTGATTAATAATTAAGTGGCACATAAACTAAGAGCAAA

CCTTTTCCATATTAGCACATCTATATTCTTTTAGTGTATCCTGTCACATACTGCATCTTCATGGTATCAT

TTACCGTTAAATGCCAAGTACCACTGCGCTTTTCAAAGCAGGGCATGCTGGATTTAAAAGAGAGTAAGGT

AATTATGTCAGGTGTCATAATGGACTACCAGCAAAAATGTGCTTTAAATAAATGTACTAATTCCGTCAGG

TAGGAATTACCT

**Tag 10: kmer assembly =** CAGCTC [C] TGACATCCTATAACCATGCATGAATGGAGC

Tag10_1F GGAGTCCTTGAGTATCAGACG

Tag10_2F CTCCCACCACTTCCATGTTG

Tag10_1R CAACATGGAAGTGGTGGGAG

Tag10_2R CGTCTGATACTCAAGGACTCC

Tag 10 SNP marked in Additional file 7

**33190 (small piece of gsdf from RNAseq):**

SF_GenWalk_33190_1F GCACGGAGGAAATTACAAGG

SF_GenWalk_33190_1R TCTGCAACCCCAAAGTGAAC

SF_GenWalk_33190_2F GTTCACTTTGGGGTTGCAGA

SF_GenWalk_33190_2R CCTTGTAATTTCCTCCGTGC

33190 is exon 4 of gsdf, see additional file 7

**Scoring Tags 2 and 10: Both scored through sequencing of PCR products**

Tag2_1F ATCAGGCCTCTGGATGGCAG

Tag2_2R AAAGCGCAGTGGTACTTGGC

Tag10_Score_1F CTTGCATCCTCCACAATCCT

Tag10_Score_1R TTCGGAGTGCTGGAAAACTT

Cycled at:

95^o^C for 3 minutes

95^o^C for 30 seconds

52^o^C for 30 seconds x35 cycles

72^o^C for 30 seconds

72^o^C for 10 minutes

**Long PCR primers:**

Tag10_1F: GTTATAGGGTCACAGCTATGCAGAGAGCTA

Unk_1R: ACCCAGTTGTCCCATCCCAGATCT

Tag2_1F: TGGAGGCTCGGGACTGCTGAAC

SF_GenWalk_33190_1R: TCTGCAACCCCAAAGTGAAC

**Sequencing of KC623942 and KC623943**

The following combinations of primers were used to sequence through the putative gsdf promoter and gene, including the sex-specific sequences, to produce the final assembled sequences. In instances where multiple forwards and reverse primers are grouped together, each combination of forward and reverse primers were amplified and sequenced. All PCR primers were also used as sequencing primers, while some primers were used solely as sequencing primers.

**Forward Group 1:**

Tag10_Score_1F CTTGCATCCTCCACAATCCT

**Reverse Group 1:**

Tag10_Score_1R TTCGGAGTGCTGGAAAACTT

**Forward Group 2**: (While some primers are marked as F and R in the same forward group, both are F. This results from the orientation at the time of design, later design reflects a corrected orientation)

Tag10_score2F ACATGGAAGTGGTGGGAGAC

Tag10_2R CGTCTGATACTCAAGGACTCC

**Reverse Group 2:**

Tag10_Score2R GGGCATCATACCCAAAAATG

Tag10_12R TGGCACGCTGTTGCTAATAC

Tag10_13R TCTGCATCAGCTTGTTCTCG

**Forward Group 3:**

Y-insert-2F CTGCACTCTCTGGATGTGGA

Tag10_12F CCCAGAAGAGCGAGAACAAG

Tag10_13F GGGCTGTTGGATTTCATTTG

**Reverse Group 3:**

GSDF1_11R GCGCAACATTTGTTCATAGGC

GSDF1_12R ATGAAAGGTGAGAGCGCAAC

**Sequencing Primers 3:** (Used in addition to PCR primers)

Y-nested-1F GTCAGAAGGCAGTGGTGTAGT

Y-nested-1R CCTTCTGACAGTGGTGTAGTG

**Forward Group 4:**

GSDF1_11F TGAACAAATGTTGCGCTCTC

GSDF1_12F TGTCTTGCAGTCATCCAAGG

**Reverse Group 4:**

Afim_GSDF_GW_1F CAGTGGCCATAGAACAGCAC

Afim_GSDF_GW_2F GATACCATCAAGCCCACCAG

**Forward Group 5:**

Afim_GSDF_GW_1R CTGGTGGGCTTGATGGTATC

Afim_GSDF_GW_2R GTGCTGTTCTATGGCCACTG

**Reverse Group 5:**

SF_GenWalk_33190_1F GCACGGAGGAAATTACAAGG

GSDF2_11R GCGAGACTTTGCTTTGGTTC

GSDF2_12R TATCCTCAGTTGGCCTTTCG

**Sequencing Group 5:**

GSDF1_13F GCAACCCCAAAGTGAACACT

All Cycled at:

95^o^C for 3 minutes

95^o^C for 30 seconds

55^o^C for 30 seconds x35 cycles

72^o^C for 1minute 30 seconds

72^o^C for 10 minutes

**Sex-specific sequence primers:**

**X-Chromosome: Primers used to produce Figure 5b/d**

X-insert-2F GTGCAGCCAAATATTGCRTA

X-insert-2R TGCTGCACTGTACCATCAAA

Cycled at:

95^o^C for 3 minutes

95^o^C for 30 seconds

55^o^C for 30 seconds x35 cycles

72^o^C for 30 seconds

72^o^C for 10 minutes

hold at 4^o^C

**Y-Chromosome: Primers used to produce Figure 5c**

Y-insert-2F CTGCACTCTCTGGATGTGGA

Y-insert-2R AATCCAAAGACAACCCAACG

Cycled at:

95^o^C for 3 minutes

95^o^C for 30 seconds

55^o^C for 30 seconds x35 cycles

72^o^C for 1 minute

72^o^C for 10 minutes

hold at 4^o^C

Dilute 1/100 and use as template in nested reaction

Y-nested-1F GTCAGAAGGCAGTGGTGTAGT

Y-nested-1R CCTTCTGACAGTGGTGTAGTG

Cycled the same as reaction 1

***gsdf*: Primers used to produce Figure 5d**

Cycled at:

95^o^C for 3 minutes

95^o^C for 30 seconds

55^o^C for 30 seconds x35 cycles

72^o^C for 30 seconds

72^o^C for 10 minutes

hold at 4^o^C
